# Identifying Priority Habitat for Conservation of the Australian Bustard Under Climate Change Scenarios

**DOI:** 10.1002/ece3.72619

**Published:** 2025-12-17

**Authors:** Saurav Lamichhane, Jill Shephard, Patricia A. Fleming

**Affiliations:** ^1^ Environmental and Conservation Sciences, Harry Butler Institute Murdoch University Perth Western Australia Australia

**Keywords:** Australian bustard, climate envelope modelling, coastal habitat loss, collision risk, renewable energy development, species distribution modelling

## Abstract

Birds are widely regarded as important indicators of environmental change, and identifying areas that are critical for their conservation is pivotal. The Australian bustard ( *Ardeotis australis* ), a wide‐ranging species of ecological and cultural significance, faces ongoing habitat modifications, yet its future distribution under changing climatic conditions remains uncertain. This study applies ensemble Species Distribution Modelling (SDM) to quantify the species' current and future habitat suitability under four climate scenarios (SSP1‐2.6, SSP2‐4.5, SSP3‐7.0, SSP5‐8.5) for 2050 and 2070. Results indicate that 69.7% of Australia is currently suitable for Australian bustards, primarily in arid and semi‐arid regions. Future projections show moderate habitat contraction, with losses mainly in coastal and semi‐arid regions, particularly in southern Australia, while inland arid areas remain relatively stable. Overlay analyses suggest that protected areas, including Indigenous Protected Areas (IPAs), will continue to support suitable habitat, reinforcing their role as long‐term climate refugia. Key environmental drivers influencing habitat suitability include precipitation seasonality (BIO15), precipitation in the coldest quarter (BIO19), and mean diurnal temperature range (BIO2), underscoring the species' reliance on predictable climatic patterns. Anthropogenic variables, particularly proximity to built‐up areas also contribute to habitat suitability, forecasting that ongoing land‐use changes may exacerbate climate‐driven habitat loss. Using estimated cleared areas (0.3 ha/MW), we also found that around 41% of under‐construction, 36% of operational, and 46% of proposed wind farms overlap with suitable habitat for the species, highlighting potential future conflict zones. This study provides the first national‐scale assessment of current and projected habitat dynamics for the Australian bustard, offering critical insights for conservation planning. Future conservation strategies should prioritise habitat connectivity, minimise anthropogenic disturbances, and integrate Indigenous‐led management approaches to ensure the species' long‐term persistence in a changing climate.

## Graphical abstract

69.7% of mainland Australia is currently suitable for Australian bustards, primarily in arid and semi‐arid regions. Central Australian bioregions exhibit extensive and interconnected suitable habitats, suggesting stable conditions across much of the continent's interior and northern areas. Habitat suitability is projected to decline in regions such as South Australia, New South Wales, and coastal areas of Queensland and the Northern Territory.

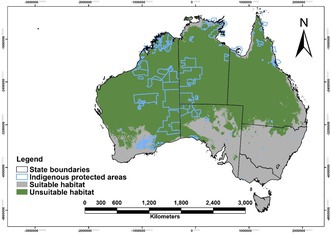

## 1 Introduction

Climate change has been shown to drive systematic shifts in species' distributions and phenology, and increase extinction risk for endemic species in biodiversity‐rich regions (Keogan et al. 2018; Manes et al. 2021; Parmesan and Yohe 2003; Pecl et al. 2017; Saino et al. 2011). A global meta‐analysis of extinction risk models indicated that climate change could threaten up to 16% of species if current emission trajectories persist, with the highest risks observed in biodiversity‐rich regions such as South America, Australia, and New Zealand (Urban 2015). Associated changes to temperature, precipitation, fire regimes, and sea level driven by climate change are expected to affect terrestrial biodiversity at all system levels, including a reduction in geographic range size and population abundance, as well as exposing many species to an increased risk of extinction (Jetz et al. 2007; Reside et al. 2014; Wardell‐Johnson et al. 2011).

Many species undergo significant shifts in population distribution in response to changes in climate (Rinnan 2018), and therefore future climate change may lead to shifts in the distribution and abundance of species (Ehrlén and Morris 2015; Wang et al. 2018). The geographical distribution of birds has also changed greatly in past decades due to climate change (Liang et al. 2021). Approximately 48% of existing bird species worldwide are known or suspected to be undergoing population declines, while only 6% show increasing trends; the remaining 39% appear stable, and 7% remain data deficient (Lees et al. 2022).

In response to these growing threats, climate change mitigation strategies—particularly the expansion of renewable energy infrastructure such as wind farms—have rapidly gained momentum (Energy Institute 2024). While these developments are essential to reduce greenhouse gas emissions and meet global climate targets, they also pose new challenges for biodiversity conservation. Energy development is now a major driver of habitat loss for birds globally and, through both direct and indirect mechanisms, can displace species, reduce habitat availability, and increase mortality from associated infrastructure (Bernath‐Plaisted et al. 2023; Loss et al. 2013; Shaffer et al. 2019). Even where suitalbe habitat remains, energy development can degrade its suitability and fragment open landscapes used by birds (Shaffer et al. 2019). Wind farms, often located in remote areas, can conflict with bird conservation, necessitating a strategic approach to avoid sensitive species concentrations (Boggie et al. 2023; Bright et al. 2008). For large, ground‐dwelling birds that inhabit these environments, such as bustard species, wind energy infrastructure may lead to habitat displacement, increased disturbance, or collision risk (Silva et al. 2023). Moreover, climate change and land‐use change often interact, jointly influencing species' distributions and amplifying conservation challenges (Mantyka‐Pringle et al. 2012).

Studying the theoretical relationship between species' geographic distribution and climate change holds significance in biodiversity conservation (Liang et al. 2021). Using research models to understand how climate change affects species' potential distribution is crucial for predicting current and future conservation challenges. Given that topographic factors and climate variability largely dictate the distribution and range of various bird species (Adhikari et al. 2023; Chhetri et al. 2018; Davies et al. 2007), it is imperative to evaluate the impact of these factors on geographic distributions and determine the degree of range alteration under forthcoming climatic conditions.

The Australian bustard ( *Ardeotis australis* ) is Australia's heaviest flying bird, and is representative of a group of terrestrial, often little‐known Australian bird species that travel widely in response to shifting habitat conditions (Ziembicki 2010). The total population is thought to exceed 10,000 but be no greater than 100,000 individuals (BirdLife International 2024). Once widespread across much of mainland Australia, bustards remain relatively common and stable in the north but have become rare or locally extinct in the south, disappearing from many areas where they were once historically recorded (BirdLife Australia 2025). Currently, they are recognised as Vulnerable in South Australia, Endangered in New South Wales, but Critically Endangered in Victoria (Atlas of Living Australia 2025). Although the species' overall population remains substantial, trends in northern Australia are also variable, with a patchy mix of declines that are only partially offset by localised increases (Garnett et al. 2011; Legge et al. 2024).

The Australian bustard is not only an ecologically important species, but also holds profound cultural significance for Indigenous Australians, featuring in Dreaming narratives, seasonal fire management practices, and as a traditional food source (Ziembicki 2010). However, the species has suffered serious declines since European colonisation, likely attributed to factors such as pesticides, fire regime changes, pastoralism, nest disturbance, habitat alteration, and predation by introduced species (Marchant and Higgins 1990; Ziembicki 2010). Climate change is likely to compound these pressures, with projected increases in temperature extremes and altered rainfall regimes further restricting resource availability. Such shifts can affect demographic performance in grassland birds, with moderate changes benefiting some species, while extreme heat, drought, and heavy rainfall reduce survival and nesting success (Maresh Nelson et al. 2024). Additionally, Australia is witnessing a rapid increase in the construction of wind turbines across the continent, with proposed wind farms accounting for a 12‐fold increase over current wind energy generation capacity (EcoGeneration 2023). Other bustard species, including the closely related Great Indian ( *A. nigriceps* ) and Kori ( *A. kori* ) bustards, are known to be at risk from collisions with wind farms and power lines (Silva et al. 2023), suggesting that this may also be a significant concern for the Australian bustard.

Despite the ecological and cultural significance of the Australian bustard, little has been done to quantify its habitat or predict its future distribution. Legge et al. (2024) recently mapped the current habitat suitability of the Australian bustard and its overlap with protected areas and bioregions, focusing on a specific region covering approximately 42% of Australia. However, no study has assessed habitat suitability nationwide or projected future changes in the species' distribution under climate change scenarios. Understanding how species' spatial distributions change under a shifting climate is essential for protecting biodiversity and formulating effective policies (Liang et al. 2021; Northrup et al. 2019; Vickery et al. 2014).

Identifying suitable habitats for the Australian bustard could be one of the major steppingstones for exploring the ecology and conservation of the species in the future. To address this gap, we applied the BIOMOD2 ensemble modelling framework, which harmonises predictions from multiple algorithms to generate consensus estimates of habitat suitability (Thuiller et al. 2009). Using bioclimatic, topographic, human‐influenced, and environmental variables, we first modelled the bustard's habitat suitability across Australia under current and future climate scenarios. We then quantified the overlap of suitable habitat with Australian states, Indigenous Protected Areas (IPAs), bioregions, and wind farm sites that are operating, under construction, and proposed.

## 2 Methods

### 2.1 Data Collection and Filtering

We extracted records of the Australian bustard from the Atlas of Living Australia (ALA; http://www.ala.org.au, accessed, December 2024), which documents individual occurrences across specific locations and times. In total, we collected 27,235 records from 1970 to 2024. These observations are contributed by a range of sources, including citizen science platforms (e.g., eBird and iNaturalist), wildlife surveys by government agencies, environmental assessments, and research projects. Given that our dataset relied solely on secondary sources and included records spanning over 50 years, rigorous data filtration was essential. We employed a multi‐step filtration process. To ensure consistency in data type, we restricted the dataset to human observation records only, excluded preserved specimens, material samples, machine observations, and other record types. We also used the ‘sf’ R package (Pebesma and Bivand 2023) to read the spatial data and the ‘dplyr’ R package (Wickham et al. 2023) to remove rows with missing coordinates, ensuring our dataset contained valid spatial information.

To further refine the dataset, we applied the ‘CoordinateCleaner’ R package (Zizka et al. 2019), which detects and removes potentially erroneous geographic records. This process included identifying and eliminating duplicate records, observations located at country centroids, institutional coordinates (e.g., museum and university locations), sea‐based records, equal coordinate errors (where longitude and latitude are identical across multiple records), and extreme outliers, reducing the final datasets to 13,654 occurrence records.

Spatial autocorrelation, which can lead to overfitting in models, can be mitigated by spatial filtering of occurrence data to enhance model performance (Elith et al. 2010). To address this, we employed spatial filtering by removing multiple points within 15 × 15 km (225 km^2^) grids, retaining only a single presence point per grid using the ‘SpThin’ R package (Aiello‐Lammens et al. 2019). This grid size was selected to reflect the species' movement ecology. Ziembicki (2010) estimated the average home range of the Australian bustard as 15.7 ± 4.13 km^2^ using the minimum convex polygon (MCP) method, and 12.35 ± 1.22 km^2^ using the 95% kernel density estimate, based on data recorded from 18 individuals (12 males and 6 females). Since the MCP method generally represents the total area used by an individual over time (Mukomberanwa et al. 2024), therefore, we selected a 15 × 15 km grid for spatial filtering as representative of the landscape scale required by the birds. After filtering, the total number of occurrence records was reduced to 2,805, providing a spatially independent dataset for modelling.

### 2.2 Pseudo‐Absence Selection and Bias Adjustment

To account for variation in survey effort across the study area and ensure that bustard absences are ecologically meaningful, we applied a two‐step approach that incorporated habitat suitability classification based on environmental envelopes at known presence locations and a correction for potential sampling bias.

We first classified the study area into low‐suitability and high‐suitability zones based on environmental variables associated with known presence locations. We extracted environmental variable values at all known presence locations and calculated a suitability index across the entire study area. This index was derived from the mean environmental characteristics across multiple predictor variables, providing a relative measure of habitat suitability for the species. We then used the median suitability value of presence locations as a threshold to divide the study area into two equal parts: low‐suitability zones (bottom 50%) and high‐suitability zones (top 50%). Pseudo‐absence points were randomly generated in low‐suitability zones (Ryeland et al. 2021).

Biases can arise from various factors, including survey locations, spatial scale, the data collected, and how these data are eventually analysed For example, in a study of butterfly data from the United States, inventory completeness was higher in areas with greater human density, and this trend became more pronounced over time (Shirey et al. 2021). The higher number of biodiversity records near urban areas can be attributed to the local densities of observers and their tendency to visit more accessible locations from their homes (Bowler et al. 2022). This potential bias toward more accessible areas also affects the distribution data, emphasising the need for careful data curation (Beck et al. 2014). Since some low‐suitability areas may also be under‐sampled, rather than truly unsuitable due to low sampling effort (Ryeland et al. 2021), we applied a sampling bias correction to account for potential survey effort discrepancies. Our ‘bias layer’ contained occurrence records of 19 other bird species with similar detectability characteristics to the Australian bustard (Table S1). These species mostly forage on the ground, are easily recognised by observers, are often seen in open or disturbed areas such as roadsides and occupy a similar distributional range to the Australian bustard. The occurrence records of these bird species were also filtered using the same method as applied to the Australian bustard occurrence records. This bias layer was created by rasterising species occurrence data. To reduce the influence of spatial clustering and overrepresented survey locations, we applied a 3 × 3 focal mean filter—equivalent to a 3 km × 3 km smoothing window. This filter size was chosen as a balance between smoothing local sampling intensity without overly generalising survey effort across the landscape. Following the bias correction process, pseudo‐absence points in areas with less than 40% of the maximum observed survey effort were removed to reduce the impact of under‐sampled regions. Barbet‐Massin et al. (2012) recommended the use of a large number (e.g., 10,000) of pseudo‐absences with equal weighting for presences and absences when using regression techniques. Phillips and Dudík (2008) also found that model performance improved with 10,000 background points in the Maxent modelling framework. The pseudo‐absence generation was repeated three times, ensuring equal weighting between presences and pseudo‐absences. To maintain 10,000 pseudo‐absence points per replicate after filtering, we randomly selected from the remaining valid points. If fewer than 10,000 valid pseudo‐absences remained after filtering, the bias threshold was relaxed to retain enough pseudo‐absences while still prioritising points from well‐surveyed regions. The final dataset included presence points (Bustard = 1), and pseudo‐absence points (Bustard = 0) that were formatted for the BIOMOD species distribution modelling framework, running 90 models, consisting of 10 algorithms, three cross‐validation replicates, and three pseudo‐absence selections. The dataset was divided into two subsets, with 70% used for model calibration (training) and 30% for validation, allowing for an assessment of the models' predictive performance.

### 2.3 Bio‐Climatic and Environmental Variables

We included elevation (DEM), slope, aspect, 19 bioclimatic variables from WorldClim v2.1 (Fick and Hijmans 2017), land use and vegetation types (including dry cropping, irrigated cropping, grasslands, and woodlands), as well as Euclidean distances to roads, water bodies, and built‐up areas in our model. The bioclimatic variables included average monthly climate data for precipitation, as well as minimum, mean, and maximum temperatures, at a spatial resolution of approximately 1 km^2^. Details of these variables are provided in Table S2. The Digital Elevation Model (DEM; from Geoscience Australia's ELVIS Portal, SRTM‐derived 3 Second Digital Elevation Models Version 1.0) (Gallant et al. 2009) was resampled to a 1 km^2^ resolution to align with other spatial layers. Slope and aspect layers were generated from the DEM using the spatial analysis tools in ArcGIS Pro 3.3.5 (ESRI 2025).

Bustards are often found along road corridors, particularly in wooded regions (Ziembicki 2010). Human infrastructure, including built‐up areas, can also influence the distribution of bird populations (Zhai et al. 2024). To analyse these effects, data on Australia's road networks and built‐up areas were obtained from Geoscience Australia and processed for analysis. As bustards also prefer open habitats, including grassland plains, low shrublands, grassy woodlands, and human‐modified areas such as croplands, golf courses, and airfields, and may also be found near watercourses, especially in arid regions (Boehm 1947; Marchant and Higgins 1990; Ziembicki 2010), we included variables such as dry cropping, irrigated cropping, distance to water resources, grasslands, and woodlands in our model. We estimated the proportion cover of dry cropping and irrigated cropping from the land use data available from Australian Land Use and Management Classification (ABARES 2024). For vegetation types, we combined major vegetation groups from the National Vegetation Information System v7.0 (DCCEEW 2024) into woodlands and grasslands (Table S2). We used surface hydrology data from Geoscience Australia to derive distance‐to‐water variables for our models. The distances to water, roads, and built‐up areas were calculated using Euclidean distance tools in ArcGIS Pro.

Beaumont et al. (2008) recommended averaging multiple climate simulations (Global Circulation Models; GCMs) rather than relying on a single future scenario for species distribution modelling to account for variability and uncertainty in climate projections. Similarly, Shrestha et al. (2018) also demonstrated that a multi‐model ensemble average provides more reliable and accurate results than individual models at both global and regional scales, as it captures variability among GCMs. Therefore, we created an ensemble of five GCMs by averaging their outputs, and the ensemble values were used as predictors. For projections of species responses to future climates, bioclimatic data were downloaded from GCMs included in the World Climate Research Programme's Coupled Model Inter‐comparison Project Phase 6 (CMIP6) ensemble (Fick and Hijmans 2017). The selection of GCMs followed recommendations from studies evaluating their skill in simulating Australian climate (Di Virgilio et al. 2022; Grose et al. 2023). We selected the models ACCESS‐CM2, CMCC‐CM2‐SR5, GISS‐E2‐1‐G, and MIROC6 to provide a comprehensive representation of current and future climatic conditions relevant to Australia:

1. ACCESS‐CM2 is one of Australia's contributions to CMIP6, which shows a better global hydrological balance, more realistic water properties (in terms of spatial distribution) and meridional overturning circulation in the Southern Ocean, but poorer simulation of the Antarctic sea ice and a larger energy imbalance at the top of the atmosphere (Bi et al. 2020). This model has also been applied in ecological studies, including projections of future habitat suitability for Australian bird species under climate change scenarios (e.g., Ryeland et al. 2021; Shephard et al. 2025).
2. CMCC‐ESM2 (one of the Centro Euro‐Mediterraneo sui Cambiamenti Climatici contributions to CMIP6) improves the representation of biogeochemical cycles, ocean dynamics, and terrestrial carbon fluxes, but has limitations in accurately simulating regional precipitation patterns and certain aspects of atmospheric circulation (Lovato et al. 2022). The model has also been used in ecological applications, including studies projecting bird distributions under future climate scenarios (e.g., Brodie et al. 2024).
3. GISS‐E2.1 (National NASA's contribution to CMIP6) features refinements in climate variability simulation, with improved representation of the Madden‐Julian Oscillation and Pacific Decadal Oscillation compared to earlier GISS models. It also enhances the simulation of the Southern Ocean climate and sea ice extent through updated atmospheric and oceanic processes, but has a slightly higher climate sensitivity (2.7°C–3.1°C) due to lower CO_2_ radiative forcing and stronger positive feedbacks (Kelley et al. 2020). Unlike some other climate models, GISS‐E2.1 has not yet been applied in studies projecting bird distributions under future climate scenarios. However, its inclusion in our ensemble modelling approach is necessary due to its improved simulation of large‐scale climate variability, which can influence key environmental drivers of species distributions, particularly in the Australian region.
4. MIROC6 (Japan's contribution to CMIP6) improves the simulation of tropical precipitation, the Madden–Julian Oscillation, and midlatitude atmospheric circulation due to the inclusion of shallow convective parameterisation and an enhanced stratospheric representation compared to earlier MIROC models, but maintains an effective climate sensitivity due to offsetting radiative forcing and climate feedbacks (Tatebe et al. 2019). The model has also been used in ecological studies, including projections of habitat suitability for various bird and plant species under future climate scenarios (e.g., Adhikari et al. 2023; Archibald et al. 2024)
5. EC‐Earth3 (Europe's contribution to CMIP6) features a flexible coupling framework, improved physical and dynamic processes, and enhanced Earth system model components, making it suitable for a wide range of CMIP6 experiments (Döscher et al. 2021). Although it has not been widely applied in bird distribution studies, EC‐Earth3 is one of the most comprehensively tested CMIP6 models and performs well in simulating coupled climate–biosphere interactions, including surface temperature, precipitation patterns, and vegetation–climate feedbacks (Döscher et al. 2021).

### 2.4 Data Analysis

To ensure comparability between current and future habitat suitability, we generated two sets of current models: (1) using only bioclimatic variables (to match the future projections) and (2) incorporating a broader set of predictors, including topographic, environmental, anthropogenic, and bioclimatic variables. The bioclimatic‐only current model (model1) was used as a baseline for comparison with future projections to account for climate‐driven changes under four different scenarios. Future climate scenarios were analysed under SSP1‐2.6, SSP2‐4.5, SSP3‐7.0, and, SSP5‐8.5. Further details about these scenarios are provided in Table S3.

All analyses were conducted in R version 4.4.2 (R Core Team 2023) using spatial data layers representing topographic, bioclimatic, environmental, and anthropogenic variables across the study region. Raster layers were standardised to identical spatial resolution and extent prior to analysis. Bioclimatic variables often exhibit a high degree of collinearity, which can lead to inaccurate and misleading model performance (Ahmad et al. 2020). Predictor variables were therefore screened for multicollinearity using Variance Inflation Factor (VIF) analysis with a threshold of VIF < 5 (Chatterjee and Hadi 2015) using the ‘usdm’ R package (Naimi et al. 2014). Variables with VIF values exceeding 5 were systematically excluded during the model construction process. The variables retained after VIF filtering for the climate‐only model (model 1) included bio2, bio3, bio9, bio15, bio18, and bio19. For the model incorporating all variables (model 2), the retained predictors were bio2, bio3, bio9, bio15, bio18, bio19, slope, aspect, elevation, distance to water bodies, distance to built‐up areas, distance to roads, dry cropping, irrigated cropping, grasslands, and woodlands.

As no single modelling algorithm consistently provides the best predictive accuracy, employing an ensemble of multiple algorithms is widely considered to achieve better accuracy (Adhikari et al. 2023; Shrestha et al. 2018; Thuiller et al. 2009). We used 10 algorithms in an ensemble approach using the BIOMOD2 package to predict the suitable habitat of the Australian bustard. The 10 algorithms used four regression methods (GLM: Generalised Linear Model, GAM: Generalised Additive Model, MARS: Multiple Adaptive Regression Splines, GBM: Generalised Boosting Model), four machine learning methods (ANN: Artificial Neural Network, RF: Random Forest, MAXENT: Maximum Entropy, SRE: Surface Range Envelope), and two classification methods (CTA: Classification Tree Analysis, FDA: Flexible Discriminant Analysis). To enhance predictive accuracy and prevent overfitting, we also adjusted model parameters to achieve an optimal level of complexity. The data were divided into training (70%) and testing (30%). The area under the curve (AUC) and true skill statistics (TSS) approaches were used to assess the effectiveness of prediction models (Thuiller et al. 2009). We chose models with TSS score > 0.60 (See Table S4) to build an ensemble from the projection outputs of the 10 algorithms by using a weighted mean approach that weights each model output according to predictive performance (TSS score) (Marmion et al. 2009).

Wind farm project data were sourced from the EcoGeneration Wind Map of Australia (EcoGeneration 2023). This map provides approximate locations, capacities, and development status (Operating, Under Construction, Proposed) of wind energy projects across Australia. Each wind farm is represented by a labelled dot indicating its approximate geographic position and corresponding attributes. Wind farm infrastructure was not included as a predictor in the SDM; instead, we modelled habitat suitability independently and assessed spatial overlap with wind farm locations after the modelling process. To extract this information, the georeferenced using known coordinate points to align it accurately with spatial data layers. Once georeferenced, the wind farm locations were digitised as point features, and attributes such as project name, capacity (in megawatts, MW), and development status were manually entered based on the map's legend and labels. Gaughan (2018) reports that while wind farms may require 1–16 ha per megawatt (ha/MW) of land due to turbine spacing, the actual land clearing required for wind energy infrastructure is significantly smaller, with approximately 0.3 ha/MW typically needed. Based on this estimate, we calculated the cleared area for each project by multiplying installed capacity by 0.3 ha/MW. This area was used to generate a circular buffer around each site. Buffers were overlaid on a binary habitat suitability raster for the Australian bustard derived through the SDM process to identify the number of suitable habitat pixels (value = 1) within each wind turbine buffer. Pixel counts were converted to area in square kilometres (km^2^) by multiplying by a factor of 0.8606 km^2^ per pixel. This conversion factor corresponds to the approximate surface area of a 30 arc‐second (~1 km) pixel at mid‐latitudes, consistent with the spatial resolution of the WorldClim Bio2 layer used in the SDM. Results were summarised by wind farm development status as either ‘Proposed’, ‘Under Construction’, or ‘Operating’.

To calculate the suitable area according to states, bioregions, IPAs, and windfarm sites, the suitable area obtained from ensemble models was intersected with these regions using the ‘Zonal Histogram’ function in ArCGIS Pro (Version 3.3.5).

## 3 Results

### 3.1 Current Habitat Distribution of Australian Bustards

**FIGURE 1:**
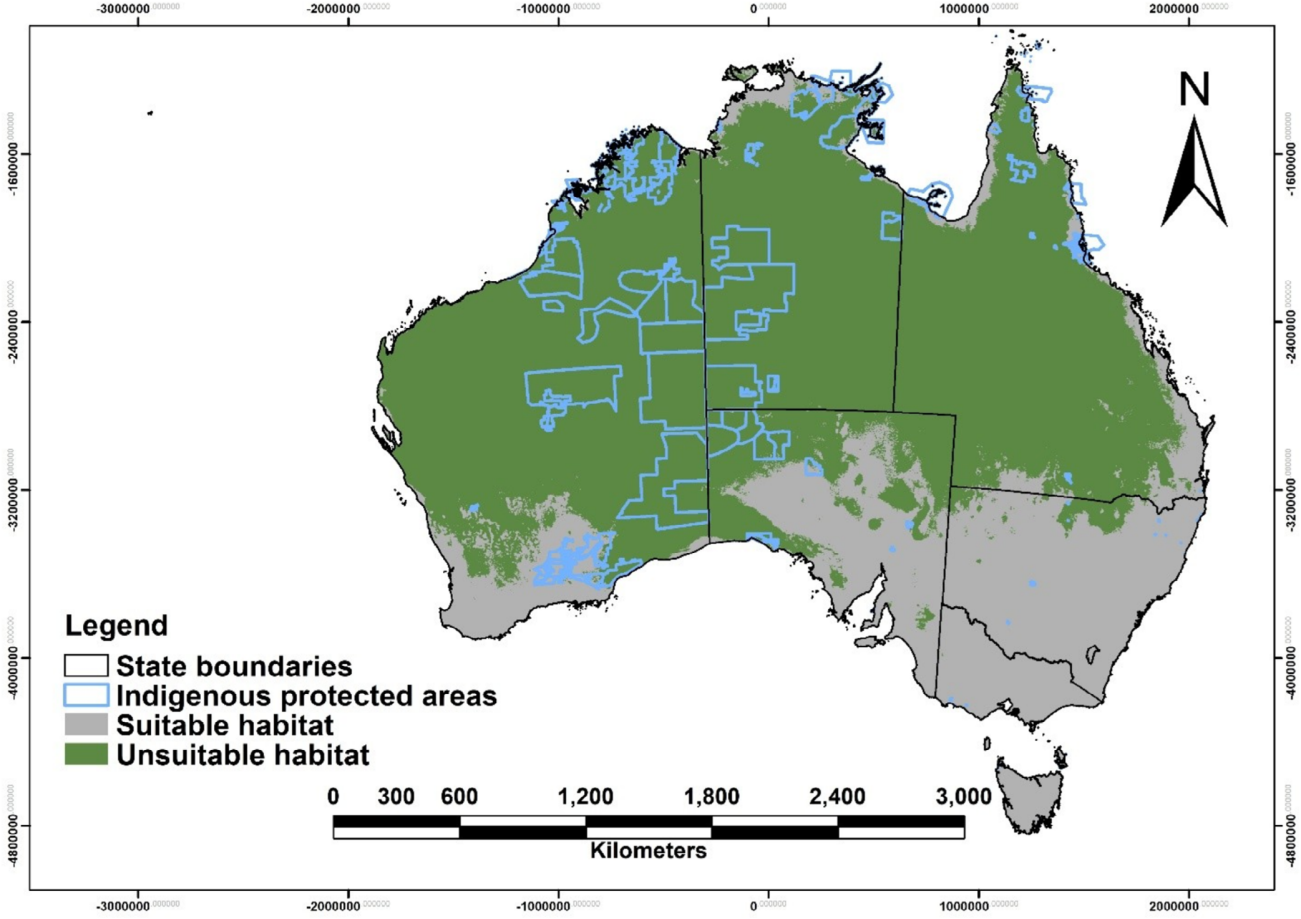
Current habitat suitability of the Australian bustard ( *Ardeotis australis* ) in Australia. Green represents suitable habitat, while grey represents unsuitable habitat. The map also includes state boundaries (black), and IPAs (outlined in light blue) for spatial context.

Our model predicted that 537,238,673 km^2^ (69.72% of Australia's total land area) is currently suitable habitat for the Australian bustard (Figure 1). State‐wise, the Northern Territory (NT) has the highest proportion of suitable habitat, with 94% of its area classified as suitable, followed by Queensland (QLD) (88%) and Western Australia (WA) (86%). South Australia (SA) has a nearly even split, with 45% suitable habitat. In contrast, New South Wales (NSW) has only 7% suitable habitat, while Victoria (VIC), Tasmania (TAS), the Australian Capital Territory (ACT), and other territories have no suitable habitat (Table S5).

Habitat suitability for the Australian bustard is highest in arid and semi‐arid bioregions (Figure S3 and Table S6), particularly in desert landscapes, open grasslands, and inland plains. Several bioregions, such as the Burt Plain (central NT), Sturt Plateau (northern NT), Tanami Desert (central NT–WA), and Channel Country (arid inland Australia), have nearly 100% suitable habitat, indicating that these regions provide optimal conditions for the species. Other semi‐arid regions, including savanna woodlands and inland floodplains, also maintain high suitability, although some variability exists due to seasonal resource availability. In contrast, high‐altitude, temperate coastal, and densely forested bioregions remain entirely unsuitable. Several regions, such as the Australian Alps (southeastern Australia), and Tasmania's highlands have 0% suitability, reflecting the bustard's avoidance of closed‐canopy vegetation, cold and high rainfall areas, and rugged terrain. Similarly, temperate coastal bioregions, including parts of the Sydney Basin (NSW) and Victorian Midlands (VIC), provide little or no suitable habitat due to fragmented landscapes and unsuitable climatic conditions.

Several IPAs in arid and semi‐arid landscapes (Figure S4 and Table S7), such as Ngurra Kayanta (Western Desert, WA), Anangu Tjutaku (central NT), and Northern Tanami (north‐central NT), have 100% suitable habitat, highlighting the role of large, intact, open landscapes in supporting bustard populations. Other IPAs in remote inland areas also maintain high suitability, ensuring long‐term habitat availability. However, IPAs in temperate, forested, and coastal regions have little to no suitable habitat. Overall, habitat suitability is highest in arid and semi‐arid landscapes across both bioregions and IPAs, while high‐altitude, temperate, and coastal regions remain largely unsuitable.

### 3.2 Model Performance

**FIGURE 2:**
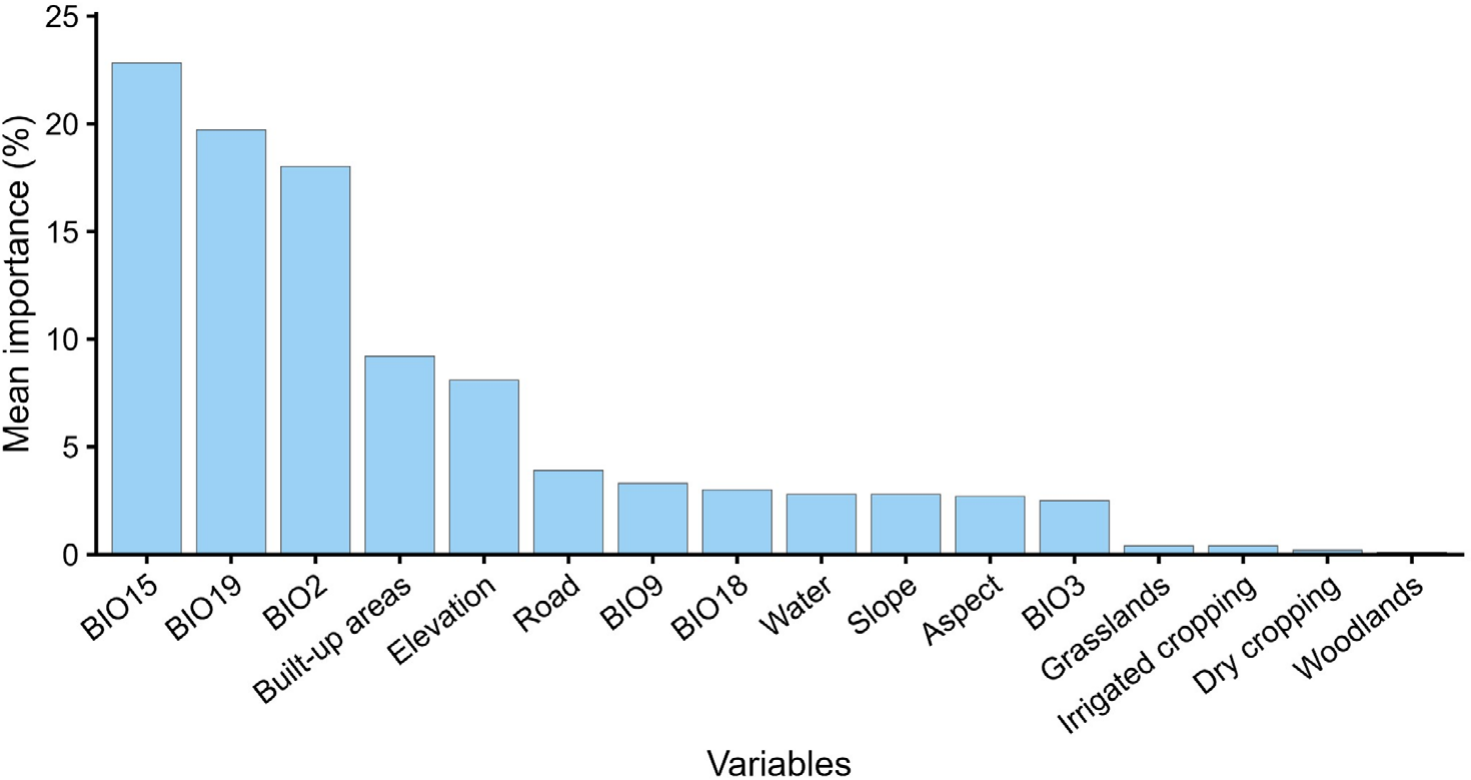
Mean variable importance for model 2 predictions of Australian bustard ( *Ardeotis australis* ) habitat suitability. Precipitation seasonality (BIO15), precipitation of the coldest quarter (BIO19), and mean diurnal temperature range (BIO2) were the most influential climatic factors, while built‐up areas and elevation were the key nonclimatic contributors.

All models were evaluated using AUC and TSS. Random Forest consistently performed the best of all the models (Figure 2), achieving the highest AUC (0.97) and TSS (0.82 for bio‐climatic only model 1, 0.79 for all‐variable model 2), indicating strong predictive ability. GBM and MAXENT also demonstrated consistent performance in both models, with AUC values around 0.88–0.89 and TSS values around 0.62–0.64, making them reliable alternatives. FDA and GLM improved in the all‐variable model 2, showing that additional environmental factors contributed to predictive performance. SRE and GAM did not appear to be effective models and lacked predictive strength for bustard distribution for both models. Full model evaluation results and performance plots are provided in Figure S1 and Table S4.

### 3.3 Variable Importance Measures

Australian bustard habitat seems to be largely influenced by bioclimatic variables (Figure 2), with precipitation seasonality (BIO15, 20.8%), precipitation in the coldest quarter (BIO19, 17.9%), and mean diurnal temperature range (BIO2, 16.4%) contributing 55.0% to the all‐variable model 2. The higher contribution of BIO15 suggests that bustards prefer areas where rainfall follows a predictable seasonal pattern. Similarly, the importance of BIO19 indicates that water availability during colder months also plays a key role in shaping their range. BIO2, which represents the difference between day and night temperatures, also ranked high in the model, suggesting that bustards may favour areas with moderate daily temperature fluctuations.

Beyond climate, distance to built‐up areas (8.36%) also had some influence on model 2, suggesting that bustards tend to avoid highly developed areas, likely due to habitat fragmentation or human disturbance (Figure S2). Elevation (7.38%) also contributed to the model, indicating preference for lowland or gently elevated terrain (Figure S1). Likewise, distance to roads (3.53%) showed a positive correlation in the model, suggesting that bustards prefer areas farther from road networks, likely due to reduced disturbance and collision risks. Land cover variables showed weaker relationships. Dry cropping and irrigated cropping had minimal influence (neutral effect), while grasslands showed a slightly positive effect on habitat suitability. Woodlands showed slightly negative effect, emphasising the known avoidance of densely vegetated regions by Australian bustard.

### 3.4 Overlap Between Wind Farm Development and Habitat Suitable for Australian Bustard

**FIGURE 3:**
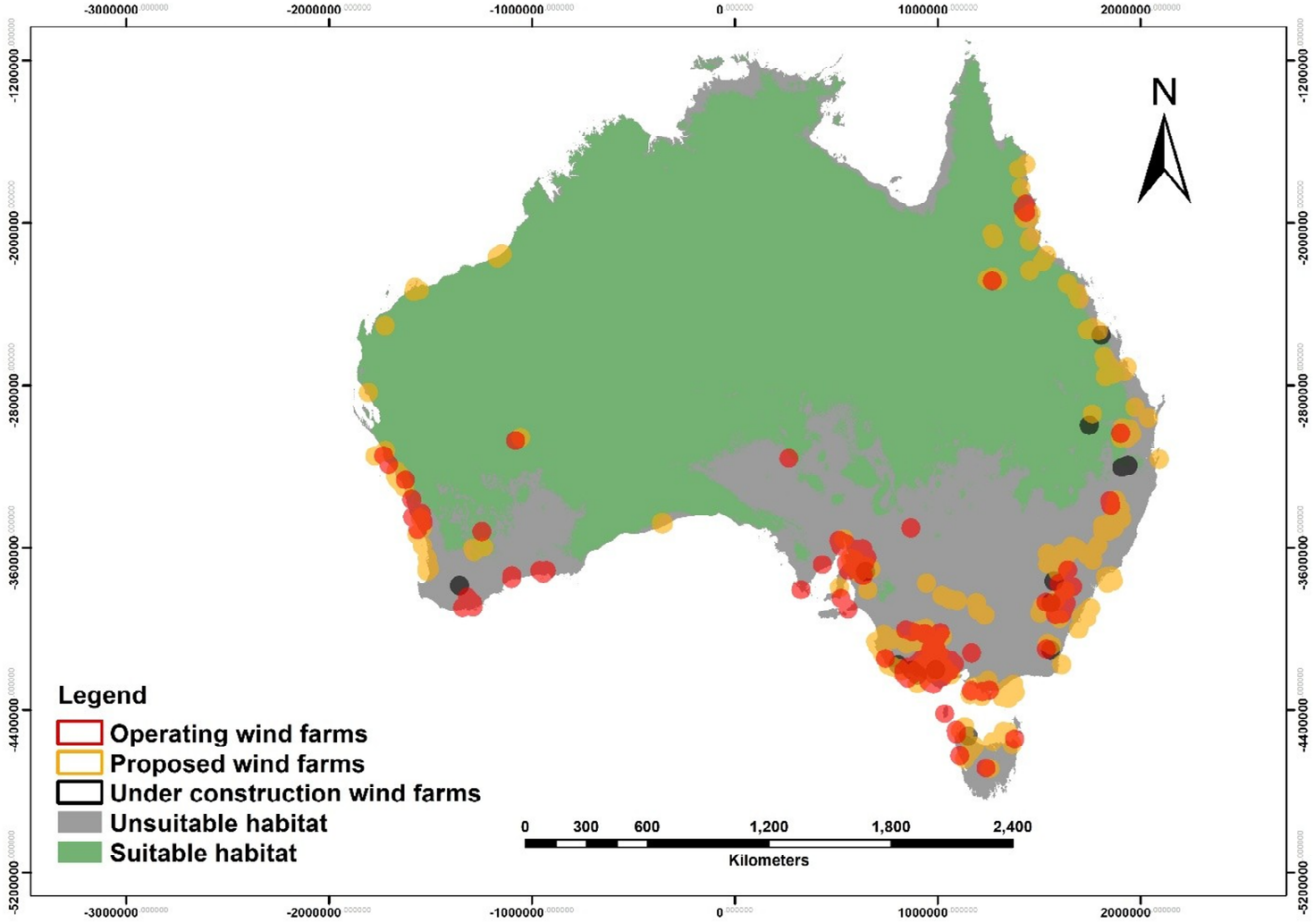
Spatial distribution of wind energy developments overlaid with Australian bustard ( *Ardeotis australis* ) habitat suitability. Green areas represent suitable habitat, while grey areas indicate unsuitable habitat. Wind farm locations are categorised by development status: operating (red), under construction (black), and proposed (orange) (EcoGeneration 2023). The majority of overlap between wind farms and suitable bustard habitat occurs in coastal regions.

There was a strong overlap between wind farm sites and suitable habitat for the Australian bustard, particularly in coastal areas. For operating wind farms, the total cleared area was estimated at 33.34 km^2^, with approximately 36% overlapping with suitable bustard habitat. Among the wind farm areas currently under construction, representing an estimated cleared area of approximately 10.43 km^2^, around 41% of this area lies within habitat classified as suitable for the Australian bustard. In contrast, proposed wind farm projects, which collectively represent about 504.75 km^2^ cleared area, are more likely to be built on areas that represent suitable habitat for the bustard, with about 46% of this area falling within the bustard's suitable habitat range (Figure 3).

### 3.5 Predicted Future Distribution and Change in the Habitat

**FIGURE 4:**
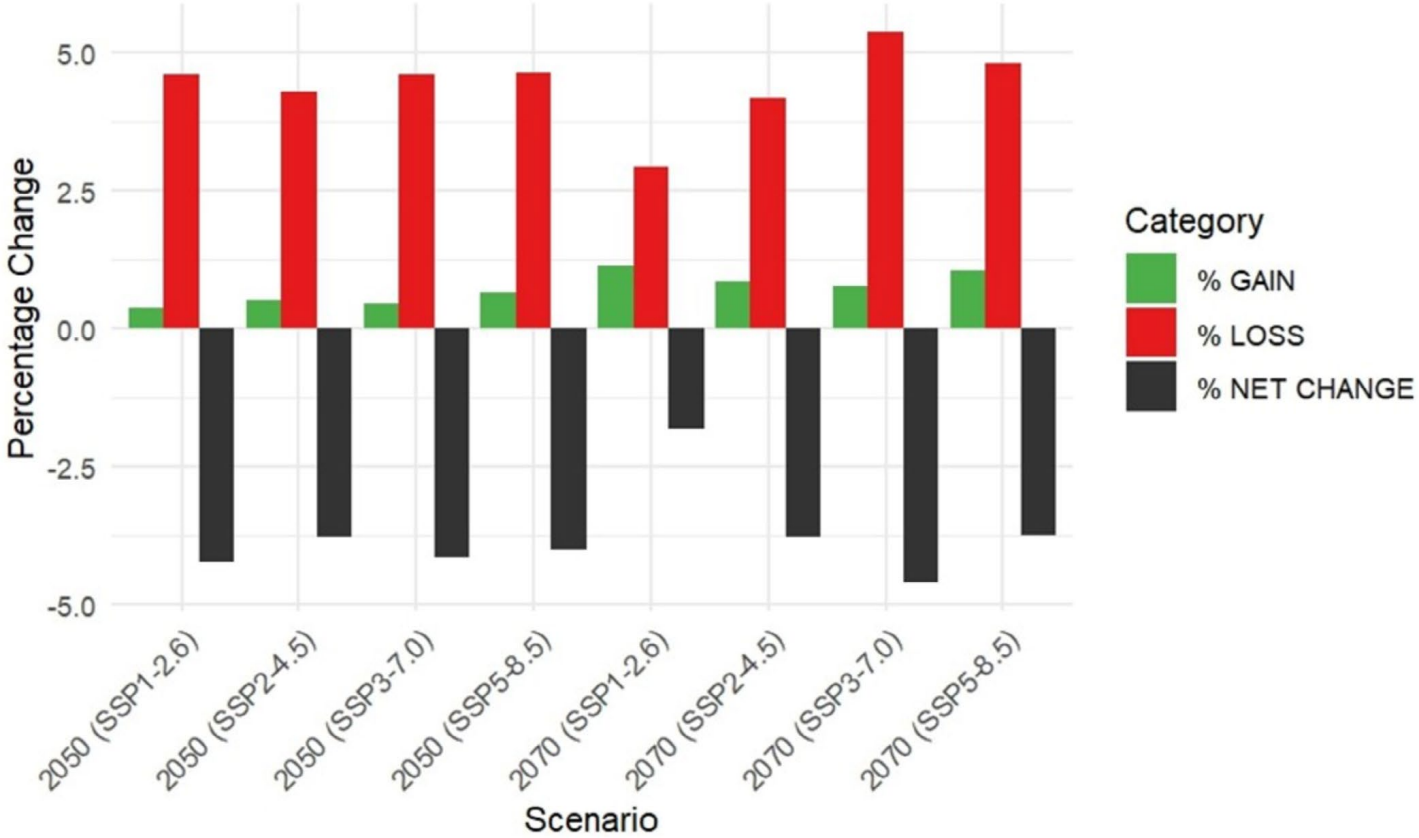
Predicted habitat changes of Australian bustard ( *Ardeotis australis* ) under future climate scenarios.

The projected habitat changes for the Australian bustard under different climate scenarios (SSPs) indicate a moderate reduction in suitable habitat across all future projections, but the extent of this loss varies slightly between scenarios (Figure 4). The highest habitat loss is observed in coastal regions, where suitability declines across all SSPs (Figure 6). The highest net habitat loss is projected under SSP3‐7.0 for the year 2070, followed by SSP1‐2.6 for the year 2050. In comparison, the SSP5‐8.5 scenarios show slightly lower habitat reductions (−3.993% in 2050 and −3.73 in 2070), despite being the highest emission scenario. SSP2‐4.5 shows intermediate habitat loss, while the lowest projected habitat loss is under SSP1‐2.6 for the year 2070. Overall, the results indicate that while climate change is expected to reduce the Australian bustard's habitat to some extent in the future, the projected loss remains relatively small across all scenarios.

**FIGURE 5:**
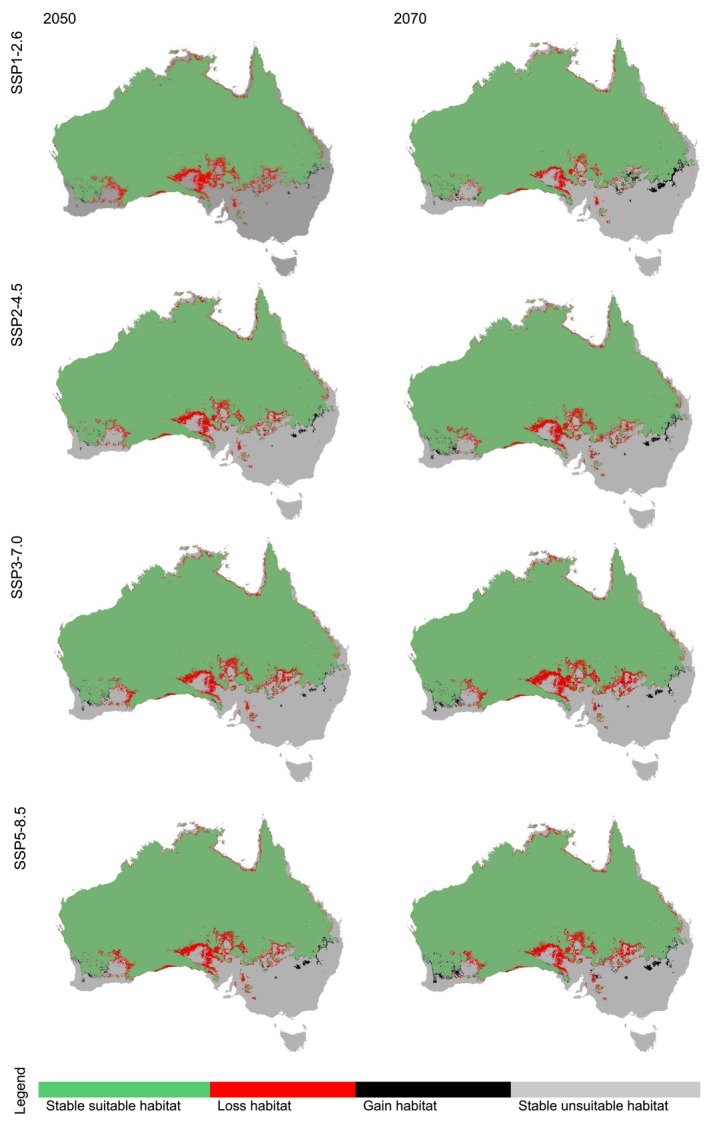
Projected habitat suitability of the Australian bustard ( *Ardeotis australis* ) in Australia for 2050 and 2070 under four climate scenarios (SSP1‐2.6, SSP2‐4.5, SSP3‐7.0, and SSP5‐8.5) indicating habitat changes primarily along coastal regions.

South Australia is projected to experience the most consistent habitat losses for the Australian bustard across all climate scenarios (Figure 5 and Table S5). By 2050, suitable habitat in SA is expected to decline by 20% under SSP1‐2.6 and by around 17% under scenarios SSP3‐7.0 and SSP5‐8.5. NSW shows a low percentage of stable suitable habitat for the Australian bustard across all scenarios,ranging between 9% and 13% in 2070 (lowest under SSP 3–7.0 to and highest under SSP1‐2.6). Corresponding habitat losses in 2070 range from 1% to 5% across scenarios. In contrast, QLD, NT and WA maintain stability with minimal losses, while VIC, TAS, the ACT, and Other Territory remain entirely unsuitable.

**FIGURE 6:**
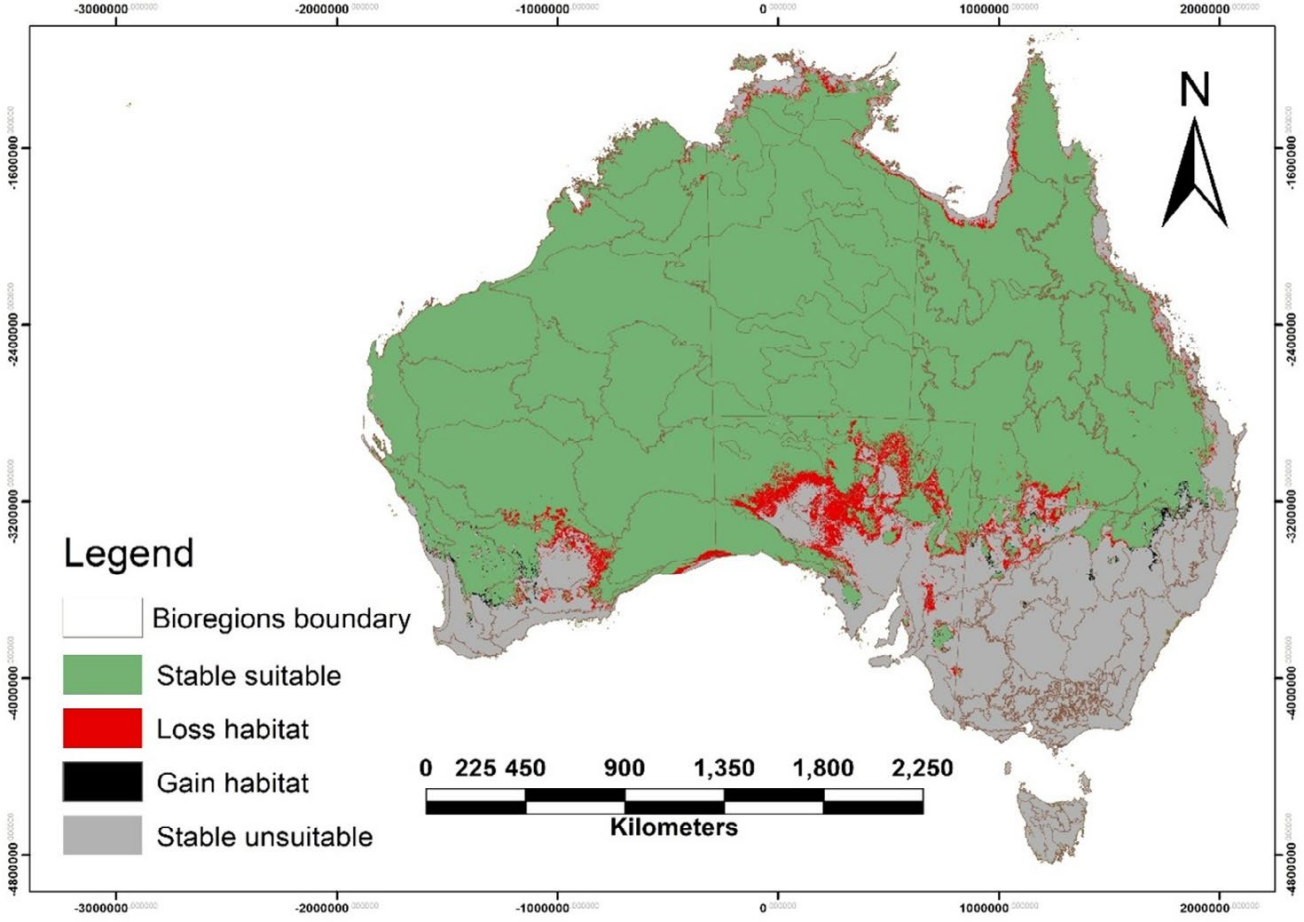
Projected habitat suitability for the Australian bustard ( *Ardeotis australis* ) under the SSP1‐2.6 climate scenario for 2050. The map delineates bioregions, with green indicating stable suitable habitat, grey representing stable unsuitable habitat, red showing habitat loss, and black depicting habitat gain. Please see Figure S3 for figures corresponding to other climate scenarios and time periods.

Projections for the Australian bustard's habitat across bioregions reveal some variations between 2050 and 2070 for all scenarios (Table S6, Figure S3 and Figure 6). Core desert bioregions (e.g., Great Sandy (north‐west WA/NT), Gibson (central WA), and Little Sandy (central‐western WA) Deserts) preserve nearly 100% of their suitable habitat even by late century, showing no climate‐driven losses. Several northern and inland bioregions also stay largely stable. For instance, the Arnhem Plateau (Top End, NT) and Brigalow Belt North (central‐eastern QLD) maintain ~85%–90% of their habitat suitability or more in future projections. Southern Semi‐Arid bioregions like the Gawler (SA) and Hampton (southern WA) regions show the largest contractions. By 2070 (high‐emission scenario), these areas could lose roughly half of their original suitable habitat. Some coastal/monsoon‐edge bioregions (e.g., Darwin Coastal in the far north NT, and Central Mackay Coast in eastern QLD) also show marked declines. These smaller peripheral regions may see over 50% reductions in suitable habitat area in worst‐case scenarios. Overall, the greatest losses tend to occur in regions that currently have more marginal habitat for the species.

**FIGURE 7:**
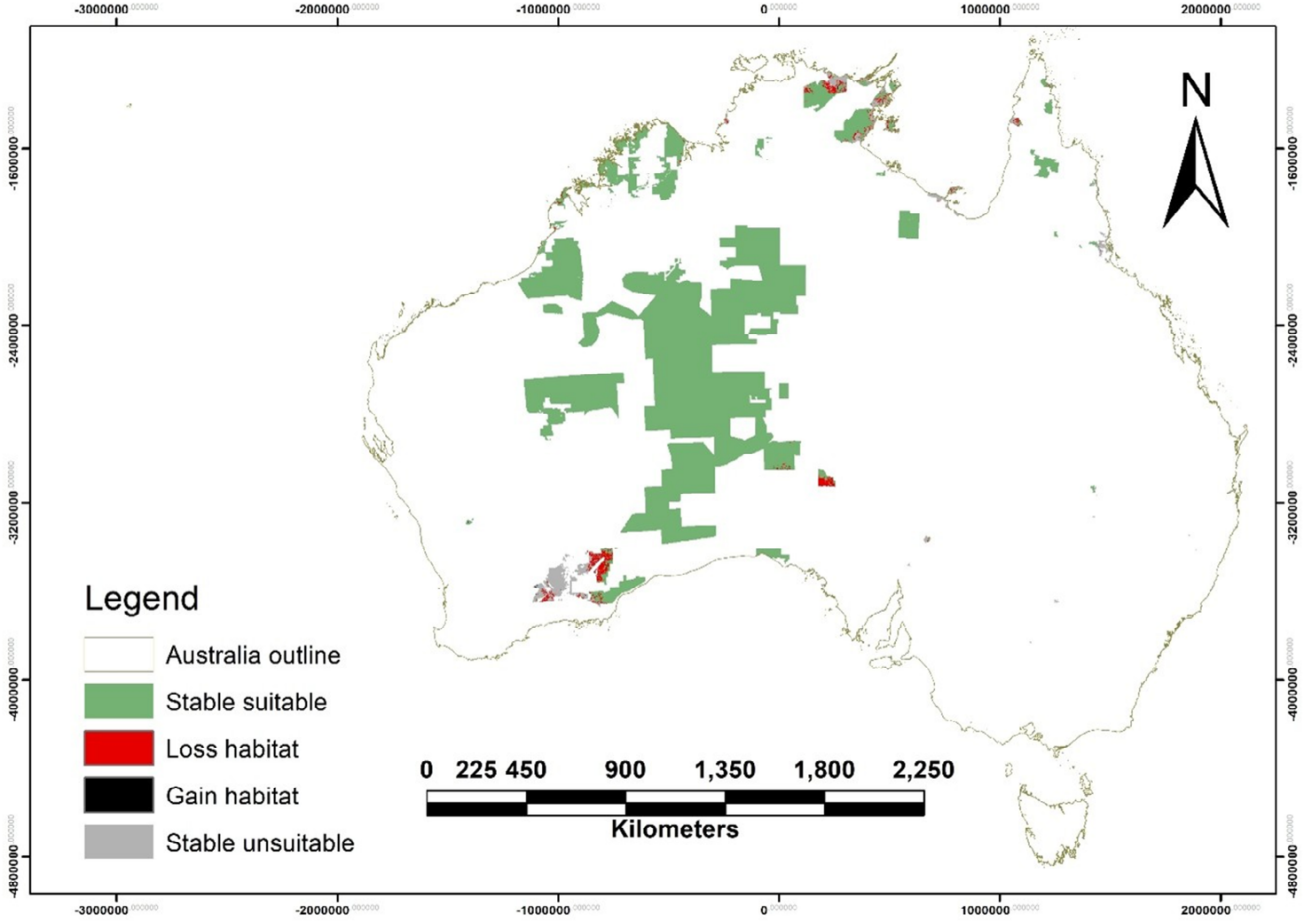
Projected habitat suitability for the Australian bustard ( *Ardeotis australis* ) within Indigenous Protected Areas (IPAs) under the SSP1‐2.6 climate scenario in 2050. The map highlights IPAs within Australia, with green representing stable suitable habitat, grey indicating stable unsuitable habitat, red showing habitat loss, and black depicting habitat gain. Please see Figure S4 for figures corresponding to other climate scenarios and time periods.

Most of the IPAs within central and northern Australia retain nearly all their current suitable habitat for Australian bustards through to 2050 and 2070 (Table S7, Figure S4 and Figure 7). For example, large IPAs such as Anangu Tjutaku (central NT) and Angas Downs (southern NT) in arid central Australia maintain 100% habitat suitability (no loss of suitable area) under both mid‐range and high‐end climate scenarios. Even under the high emission scenarios, many IPA landscapes serve as refugia where conditions remain favourable. The Northern Tanami (north‐central NT), Uunguu (northern Kimberley, WA), and other IPAs covering intact savanna or desert habitats persist as extensive suitable environments for the bustard. These areas will likely support core breeding and foraging grounds when surrounding unprotected lands might become less hospitable. A smaller number of IPAs located at the periphery of the bustard's current range do face some habitat losses. For instance, IPAs in more southerly or coastal locations (such as Ngadju in southern WA) are projected to lose a substantial portion of suitable habitat by 2070.

## 4 Discussion

The Australian bustard's habitat suitability, as modelled in this study, underscores its strong association with Australia's arid and semi‐arid ecosystems. Model performance was high, driven mainly by rainfall seasonality, cold‐season precipitation, and diurnal temperature range. Future projections suggest moderate habitat losses, concentrated in coastal and southern areas, while central deserts and Indigenous Protected Areas remain stable refugia. Overlap with wind farm developments was greatest in coastal regions, where nearly half of proposed projects intersect suitable habitat.

Nearly 70% of Australia's landmass was classified as suitable for the bustard, particularly the arid and semi‐arid regions of the NT, WA, and QLD. This represents the observational data that report the highest bustard occurrence in the Great Sandy, Victoria, and Tanami Deserts, as well as in mid‐NT and eastern‐central and western QLD (Marchant and Higgins 1990), further supported by aerial surveys conducted by Grice et al. (1986). This widespread distribution is indicative of the bustard's ability to exploit resource‐scarce environments, a trait also observed in other bustard species such as the Great bustard *Otis tarda* and the Houbara bustard *Chlamydotis undulata* , which exhibit flexible habitat use in response to seasonal fluctuations in food availability (Hingrat et al. 2007; Palacin and Alonso López 2008).

The inclusion of elevation in our models captured the bustard's preference for open, lowland habitats, often avoiding hilly regions (Marchant and Higgins 1990; Ziembicki 2010). These results align with previous ecological assessments describing bustards as habitat generalists within dryland environments but specialists in avoiding forested and rugged terrains (Ziembicki 2010). Recent findings by Legge et al. (2024) further support these patterns, highlighting that bustard detection rates have been highest in the northern and western deserts, particularly in bioregions with stable, high average temperatures, and long unburnt areas. The predominance of these regions in sustaining bustard habitat also highlights the species' reliance on lowland, open landscapes with low human population density, where visibility is high, and disturbance is minimal. Similar findings have been reported for the Little bustard *Tetrax tetrax* , which prefers open, undisturbed landscapes on the Iberian Peninsula that allow for an unobstructed long‐distance visual range (Silva et al. 2004).

The near‐total absence of suitable habitat in Tasmania, Victoria, and heavily forested or elevated bioregions (e.g., Australian Alps NSW/VIC, Jarrah Forest WA) reflects the species' known avoidance of densely vegetated, cold, and high‐altitude landscapes. This is consistent with previous research that highlights that the Australian bustard tends to avoid dense forests, high‐altitude areas, and temperate coastal regions, reflecting its reliance on sparse vegetation for foraging and display behaviours (Marchant and Higgins 1990). Such avoidance behaviour is also consistent with other ground‐dwelling birds that depend on large ground patches with short vegetation, uninterrupted landscapes for movement and foraging (Leal et al. 2019; Li et al. 2023).

### 4.1 Key Climate and Environmental Drivers of Australian Bustard Distribution

Precipitation has long been recognised as a major driver of species richness and distribution of bird species (Liang et al. 2020). Our models also show that precipitation seasonality (BIO15) and precipitation in the coldest quarter (BIO19) were the most influential predictors of Australian bustard habitat suitability. The strong influence of BIO15 suggests that bustards prefer areas with predictable rainfall patterns, which likely correspond to more reliable food availability. This is consistent with findings by Ziembicki (2010), who reported that bustard breeding in arid regions is opportunistic and highly dependent on rainfall events, whereas in northern Australia, males begin displaying before the onset of monsoonal rains, possibly in anticipation of increased productivity.

The importance of precipitation in the coldest quarter (BIO19) further supports the importance of seasonal water availability in shaping bustard habitat selection. Irregular widespread movements in bustards are often dictated by climatic conditions, with inland droughts forcing birds toward coastal regions, while significant rainfall events lead to sudden population increases in previously dry areas (Marchant and Higgins 1990; Ziembicki 2010). Such seasonal dependence on rainfall also explains why bustards are less common in temperate, high‐altitude regions, where precipitation patterns are mostly less predictable or occur at times of the year that do not align with their ecological needs. Rainfall has also been noted to influence reproduction behaviour of other ground‐dwelling birds such as Emu ( *Dromaius novaehollandiae* ), with egg production thought to increase in response to rainfall prior to incubation (Pople et al. 1991; Ryeland et al. 2021). Precipitation in the coldest quarter is therefore likely to reflect food availability for these arid‐adapted bird species.

The strong influence of mean diurnal temperature range (BIO2) in the habitat suitability model suggests that Australian bustards avoid areas with extreme daily temperature fluctuations, likely due to physiological limitations that constrain their ability to thermoregulate or forage efficiently. Instead, they appear to favour regions with more stable daily temperatures, where activity patterns can be sustained with reduced thermal stress. Ziembicki (2010) highlights how bustards adjust their behaviour in response to seasonal temperature changes, noting that males increase foraging activity in the early dry season to build energy reserves before engaging in energetically demanding display behaviour later in the season. These behavioural shifts likely reflect an adaptive response to thermal and energetic constraints, supporting the model's indication that temperature stability plays a key role in bustard habitat selection. In contrast, vegetation type showed a comparatively weaker influence in the model. Grassland, woodland, or farming practices were less important predictors of Australian bustard occurrence than the other variables included. As bustards undertake irregular, long‐distance movements in response to rainfall‐driven resource pulses (Marchant and Higgins 1990; Ziembicki 2010), they are less likely to show consistent associations with vegetation types.

### 4.2 Bustards Avoid Disturbed Habitats

Beyond climate, the models underscore avoidance of built‐up areas, emphasising the species' avoidance of anthropogenic disturbance. This finding mirrors global trends in animal habitat fragmentation, where human infrastructure displaces large‐bodied, mobile species due to reduced habitat connectivity (Greco et al. 2025). Similar observations were made by Mi et al. (2017) for Great bustards in Cangzhou, China, where the probability of the species' occurrence increases with distance from residential areas, and habitat suitability stabilises beyond 1–2 km. Avoidance of infrastructure such as roads and buildings is also a strong driver of Great bustard presence in Spain, as they are affected by land‐use changes in the area (Lopez‐Jamar et al. 2011). Similar relationships between habitat suitability and urbanisation have also been observed in the Little bustard ( *Tetrax tetrax* ), where species abundance declines with increasing proximity to urban areas (Arroyo and Casas Arenas 2022).

However, the relationship between bustards and human‐modified landscapes is not static. Ziembicki (2010) documented an initial attraction of Australian bustards to recently cleared agricultural or infrastructural areas, likely driven by ephemeral resource pulses (e.g., insect outbreaks in disturbed soils or accessible foraging in open fields), but with the long‐term pattern ultimately being population declines in some regions. Similarly, Arroyo and Casas Arenas (2022) demonstrated that while foraging habitat suitability initially influences Little bustard abundance, its significance weakens as urban encroachment increases; and when urban areas exceed 5% of the landscape, habitat suitability alone no longer predicts abundance. This suggests that land conversion may create temporary foraging opportunities, but as habitat fragmentation progresses, it reduces access to large, open landscapes that are crucial for bustard survival.

The extent and condition of land use change will therefore play a crucial role in shaping the long‐term availability of suitable habitat for species like the Australian bustard (Boehm 1947; Marchant and Higgins 1990). Most Australians live close to the coast, putting immense pressure on coastal ecosystems (Cresswell et al. 2021). According to the State of the Environment (2021) report, Australia's landcover is constantly changing due to urban expansion, agricultural intensification, and infrastructure development, with SA and NSW among the most heavily affected regions (Cresswell et al. 2021). Native vegetation provides essential ecosystem services—including habitat stability, resource availability, and connectivity—that are critical for species that rely on large, open landscapes (Keith 2017). The Human Footprint Map for Australia (2013) further highlights that SA and NSW contain some of the most modified landscapes, with extensive vegetation clearing and land‐use conversion reducing the availability of intact habitats (Williams et al. 2020).

Although generalist species like the Australian bustard can persist in modified environments, habitat fragmentation reduces landscape connectivity, movement corridors, and access to critical resources. The decline in native vegetation extent, particularly in woodland and grassland ecosystems, suggests that land‐use intensification is creating barriers that may limit dispersal and increase population isolation (Cresswell et al. 2021). Unlike the relatively intact landscapes of northern and central Australia, which remain largely undisturbed, the fragmented nature of SA and NSW landscapes—as documented by the Human Footprint Map of Australia (2013) (Williams et al. 2020) and the State of the Environment Report (2021) (Cresswell et al. 2021)—reflects long‐standing degradation that may be exacerbating projected habitat losses. Australia's Environment Report (2023) (van Dijk et al. 2024) further confirms this trend, documenting continued declines in vegetation cover and above‐average fire activity in both states, with NSW experiencing the largest drop in environmental condition score nationally. Our habitat suitability projections support these observations, indicating that regions already identified as ecologically degraded are also likely to experience significant future declines in bustard habitat under future climate scenarios. Restoring degraded landscapes in SA and NSW and enhancing connectivity between fragmented areas will be crucial for maintaining the long‐term population stability of Australian bustard across these states.

### 4.3 Habitat at Risk From Wind Energy Development

Although wind energy offers a sustainable alternative to fossil fuels, its rapid expansion has raised increasing concern over potential risks to birds (Bretagnolle et al. 2025). The rise in the number of wind turbines has led to a growing concern about habitat loss, as well as bird and bat collisions (Bose et al. 2020; Marques et al. 2014). In addition to the direct impacts of collision strikes, wind turbines indirectly disturb birds by affecting their breeding (Busch et al. 2017), habitat use (Peschko et al. 2020) and migration patterns (Kumara et al. 2022; Marques et al. 2020; Masden et al. 2009). A wide range of factors can influence bird collisions with wind turbines, including species characteristics (morphology, phenology, behaviour, or abundance), site (landscape, flight paths, food availability, and weather), and wind farm features (turbine type and configuration and lighting) (Busch et al. 2017; Kumara et al. 2022; Marques et al. 2014, 2020; Masden et al. 2009; Peschko et al. 2020). There is a growing concern among the industry and scientific community due to high bird fatality rates associated with several wind farms (Marques et al. 2014). It is crucial to consider and mitigate the adverse effects on wildlife as wind energy development projects rapidly expand worldwide (Murgatroyd et al. 2021).

Consequently, the development of wind farms within key bustard habitats may introduce additional pressures, including disturbance, displacement, and elevated collision risk. Although specific risks to the Australian bustard from wind farms have not yet been thoroughly studied, global studies on other bustard species (Janss and Ferrer 2000; Marques et al. 2021; Silva et al. 2023, 2014) suggest possible threats, particularly from collisions with wind turbines and associated energy infrastructure such as powerlines. Bustards, being ‘heavy fliers’ with limited frontal visual fields, are particularly susceptible to mortality at powerlines (Silva et al. 2023). The strong overlap between windfarm sites and Australian bustard habitat observed in our study raises concern about the potential adverse impacts on the species. Site‐specific studies to monitor bustard presence, movement, and behaviour around existing wind farm sites are necessary. For proposed developments, incorporating site‐specific habitat suitability modelling into early planning may help avoid placing new infrastructure within key bustard habitat.

### 4.4 Future Climate Projections

Species that are highly mobile and have generalist habitat preferences tend to be less vulnerable to climatic change than less mobile or specialist species (Ryeland et al. 2021; Warren et al. 2001). Generalist species can thrive in a variety of habitat types within a landscape and are generally less impacted by habitat fragmentation compared to specialist species, which are more dependent on one or few habitat types (Brouat et al. 2004; Devictor et al. 2008). Our findings support these statements, as the highly mobile and generalist Australian bustard appears relatively less affected by different climate change scenarios, with only moderate reductions in suitable habitat projected. The continued availability of habitat, particularly in arid and desert bioregions, suggests that its generalist nature provides some resilience to climate‐driven habitat changes. However, the projected habitat reductions in SA, NSW, and coastal areas of QLDland and Northern Territory indicate that mobility and habitat flexibility do not guarantee uniform resilience across the species' range.

### 4.5 An Important Role for Indigenous‐Led Conservation Into the Future

Projected habitat changes across bioregions and IPAs suggest a mixed outlook for landscape connectivity. The central Australian bioregions remain suitable and adjoin one another; consequently, a broad connected range of suitable habitat will be retained across much of the continent's interior and north. This contiguous block of habitat—spanning multiple neighbouring bioregions and IPAs—should allow bustards to move freely across large landscapes, which is positive for maintaining gene flow between populations. Habitat loss in outlying bioregions may lead to fragmentation of peripheral populations. For states like SA, where overall habitat suitability is only 45%, certain IPAs, such as Kalka‐Pipalyatjara and Walalkara, provide highly favourable habitat for the species. Prioritising conservation efforts within these IPAs can help sustain bustard populations in an otherwise marginally suitable region. As coastal and southern pockets (e.g., parts of the northern coastline and southern semi‐arid zones) become unsuitable, any bustard populations in those areas could be isolated or forced to relocate. Our findings therefore underscore the ecological value of IPAs as conservation refugia, particularly in remote, arid regions where land‐use intensity is low. The strong alignment between highly suitable bustard habitat and IPAs presents an opportunity for collaborative conservation efforts, integrating traditional ecological knowledge with scientific monitoring, as evidenced by Indigenous ranger‐led programs across central Australia (Legge et al. 2024).

### 4.6 Implications for Conservation and Management

1. The Australian bustard's habitat remains largely stable across arid and semi‐arid bioregions, with current and future projections highlighting these areas as key strongholds for the species.
2. Conservation investments should be directed to regions where bustard populations have a higher likelihood of persistence, such as the Northern Territory, Western Australia, and Queensland, or the priority IPAs in South Australia.
3. The influence of built‐up areas suggests that urbanisation and infrastructure expansion pose threats to bustards. Maintaining habitat corridors between stable bioregions and restricting development near key bustard habitats could mitigate disturbance effects.
4. Wind energy developments overlapping with suitable habitat for the Australian bustard indicate potential future conflict zones that require careful assessment and conservation planning.
5. Many IPAs overlap with high‐suitability habitats, presenting an opportunity to collaborate with Indigenous communities on conservation strategies, including around fire regime management, sustainable hunting practices, and habitat restoration. Expanding bustard monitoring programs within IPAs to assess population trends and habitat dynamics in collaboration with Indigenous people can further enhance bustard conservation, ensuring long‐term protection.
6. Although climate change is expected to moderately impact habitat suitability, proactive measures such as monitoring population shifts, restoring degraded semi‐arid grasslands, and maintaining climate refugia will be crucial for future‐proofing bustard conservation.
7. Future research integrating telemetry data and fine‐scale habitat assessments will further improve our understanding of bustard habitat dynamics and inform targeted conservation actions.

## Author Contributions

**Saurav Lamichhane:** conceptualization (equal), data curation (lead), formal analysis (lead), methodology (lead), resources (equal), validation (equal), visualization (equal), writing – original draft (lead), writing – review and editing (equal). **Jill Shephard:** conceptualization (equal), investigation (equal), project administration (equal), supervision (equal), visualization (equal), writing – original draft (equal), writing – review and editing (equal). **Patricia A. Fleming:** conceptualization (equal), investigation (equal), project administration (equal), supervision (equal), validation (equal), visualization (equal), writing – original draft (equal), writing – review and editing (equal).

## Conflicts of Interest

The authors declare no conflicts of interest.

## Supporting information


**Appendix S1:** ece372619‐sup‐0001‐AppendixS1.docx.

## Data Availability

The dataset supporting this study is available for review via the following Zenodo link: https://zenodo.org/records/16418083?preview=1&token=eyJhbGciOiJIUzUxMiJ9.eyJpZCI6ImI0ZjY5MTNhLWVhNWItNGY1NS05NGVhLTJkODU1NjEzYWI0YSIsImRhdGEiOnt9LCJyYW5kb20iOiJhMzg0Y2NiMGY0NTExOTJkMWMxZDliNjFiODg3Y2IyYiJ9.3sNeyW‐P‐8ukcbmMfI9N0QnX_dMIc1cYYVSXABvccqNdj9qpTDV7f89lix6fY5TON_eEMtiTs4Ho6XiNUnw61w.
