# Correction to “Identifying Priority Habitat for Conservation of the Australian Bustard Under Climate Change Scenarios”

**DOI:** 10.1002/ece3.73646

**Published:** 2026-05-05

Lamichhane, S., J. Shephard, and P. A. Fleming. 2025. “Identifying Priority Habitat for Conservation of the Australian Bustard Under Climate Change Scenarios.” *Ecology and Evolution* 15, no. 12: e72619. https://doi.org/10.1002/ece3.72619.

In Section 3.1 Current Habitat Distribution of Australian Bustards, the habitat area was incorrectly reported due to a unit conversion error (hectares vs. km^2^). The text “537,238,673 km^2^ (69.72% of Australia's total land area)” was incorrect. This should have read: “5,362,879 km^2^ (69.72% of Australia's total land area)”.

This correction does not affect any analyses, results, or conclusions of the study.

We apologize for this error.

