# Supplementary material for: Identifying Priority Habitat for Conservation of the Australian Bustard Under Climate Change Scenarios
Source: Ecol Evol. 2025 Dec 17;15(12):e72619. doi: 10.1002/ece3.72619 (PMC12711596; doi:10.1002/ece3.72619)
Supplement: Supplementary file 1 — Appendix S1: ece372619‐sup‐0001‐AppendixS1.docx. [file ECE3-15-e72619-s001.docx]

**Identifying priority habitat for conservation of the Australian bustard under climate change scenarios**

Table S1 Bird species used to create a bias layer reflecting sampling efforts across Australia

| S. N | Common name | Scientific name | Included Occurrences |
| --- | --- | --- | --- |
| 1. | Brolga | *Antigone rubicunda* | 33341 |
| 2. | Emu | *Dromaius novaehollandiae* | 52381 |
| 3. | Bush stone-curlew | *Burhinus grallarius* | 5659 |
| 4. | Australian bushturkey | *Alectura lathami* | 22171 |
| 5. | Spinifex pigeon | *Geophaps plumifera* | 6500 |
| 6. | Ground cuckoo-shrike | *Corancina maxima* | 22171 |
| 7. | Cape barren goose | *Cereopsis novaehollandiae* | 5172 |
| 8. | Black-necked stork | *Ephippiorhynchus asidticus* | 14978 |
| 9. | White necked heron | *Ardea pacifa* | 4231 |
| 10. | Pied heron | *Egretta picata* | 12204 |
| 11. | Stubble quail | *Coturnix pectoralis* | 13552 |
| 12. | Wedge-tailed eagle | *Aquila audax* | 100532 |
| 13. | Australian pratincole | *Stiltia isabella* | 6523 |
| 14. | Major Mitchell’s cockatoo | *Lophochroa leadbeateri* | 6787 |
| 15. | Australian magpie goose | *Anseranas semiplamata* | 54277 |
| 16. | Little crow | *Corvus bennetti* | 16204 |
| 17. | Mallefowl | *Leipoa ocellata* | 3671 |
| 18. | Plains-wanderer | *Pedionomus torquatus* | 352 |
| 19. | Beach stone curlew | *Esacus magnirostrics* | 11304 |

Table S2 Variables used in the modelling. Variables with an * are those that were included in climate only model

| Types | Variables | Code | Resolution | Source |
| --- | --- | --- | --- | --- |
| Bio-Climatic Variables | Annual Mean Temperature | bio 1 | 30 arc-seconds (~1 km²) | WorldClim (www.worldclim.com) |
|  | Mean Diurnal Range [Mean of monthly (Max Temperature - Min Temperature)] | bio 2 * | 30 arc-seconds (~1 km²) |  |
|  | Isothermality (BIO 2/BIO 7) (*100) | bio 3 * | 30 arc-seconds (~1 km²) |  |
|  | Temperature Seasonality (Standard Deviation*100) | bio 4 | 30 arc-seconds (~1 km²) |  |
|  | Max Temperature of Warmest Month | bio 5 * | 30 arc-seconds (~1 km²) |  |
|  | Min Temperature of Coldest Month | bio 6 | 30 arc-seconds (~1 km²) |  |
|  | Temperature Annual Range (BIO 5-BIO 6) | bio 7 | 30 arc-seconds (~1 km²) |  |
|  | Mean Temperature of Wettest Quarter | bio 8 | 30 arc-seconds (~1 km²) |  |
|  | Mean Temperature of Driest Quarter | bio 9 | 30 arc-seconds (~1 km²) |  |
|  | Mean Temperature of Warmest Quarter | bio 10 | 30 arc-seconds (~1 km²) |  |
|  | Mean Temperature of Coldest Quarter | bio 11 | 30 arc-seconds (~1 km²) |  |
|  | Annual Precipitation | bio 12 | 30 arc-seconds (~1 km²) |  |
|  | Precipitation of Wettest Month | bio 13 | 30 arc-seconds (~1 km²) |  |
|  | Precipitation of Driest Month | bio 14 | 30 arc-seconds (~1 km²) |  |
|  | Precipitation Seasonality (Coefficient of Variation) | bio 15* | 30 arc-seconds (~1 km²) |  |
|  | Precipitation of Wettest Quarter | bio 16 | 30 arc-seconds (~1 km²) |  |
|  | Precipitation of Driest Quarter | bio 17 | 30 arc-seconds (~1 km²) |  |
|  | Precipitation of Warmest Quarter | bio 18 * | 30 arc-seconds (~1 km²) |  |
|  | Precipitation of Coldest Quarter | bio 19 * | 30 arc-seconds (~1 km²) |  |
| Human Infrastructures | Distance to built-up area | Dist_to_built | 30 m | Geo-Science Australia |
|  | Distance to road | Dist_to_road | 30 m |  |
| Topographic Variables | Elevation | Dem | 1 arc-second (~30 m) | Gallant et al., 2009 |
|  | Slope | Slope | 1 arc-second (~30 m) |  |
|  | Aspect | Aspect | 1 arc-second (~30 m) |  |
| Environmental Variables | Dry cropping land | Dry_cropping | 250 m | [**https://www.abs.gov.au/statistics/ environment/**](https://www.abs.gov.au/statistics/environment/) |
|  | Irrigated cropping land | Irrigated_cropping | 250 m |  |
| Environmental Variables | Grasslands (Tussock grasslands, Hummock grasslands, other grasslands and Rushlands, Chenopods shrublands | Grasslands | 100 m | [**https://fed.dcceew.gov.au/datasets 5e70b5afc36a4c458a2cceb313eb3889/about**](https://fed.dcceew.gov.au/datasets5e70b5afc36a4c458a2cceb313eb3889/about) |
|  | Woodlands (Eucalypt woodlands, Eucalypt open woodlands, Tropical Eucalypt woodlands, Acacia open woodlands, Mallee woodlands, Other open woodlands, Mallee open woodlands) | Woodlands | 100 m |  |

Table S3 Summary of SSP climate scenarios, and their key characteristics

| **Aspect** | **SSP126 (Low)** | **SSP245 (Intermediate)** | **SSP370 (High** | **SSP585 (Extreme)** |
| --- | --- | --- | --- | --- |
| **Description** | **Sustainability-focused pathway**: Low emissions and strong global cooperation for climate mitigation. | **Middle-of-the-road pathway**: Moderate emissions with some mitigation policies but uneven efforts globally. | **High challenge to mitigation**: High emissions due to moderate climate policies and high dependence on fossil fuels. | **Fossil-fuel intensive pathway**: No significant climate policies, resulting in the highest emissions and most extreme impacts. |
| **Global Temperature Increase** (2100) | ~1.8°C above pre-industrial levels. | ~2.7°C above pre-industrial levels. | ~3.9°C above pre-industrial levels. | ~4.4°C above pre-industrial levels. |
| **Radiative Forcing in 2100** | ~2.6 W/m² | ~4.5 W/m² | ~7.0 W/m² | ~8.5 W/m² |
| **Key Drivers** | - Strong climate policies. - Renewable energy adoption. - Low population growth. - Global cooperation on sustainability. | - Moderate mitigation efforts. - Continued use of fossil fuels alongside renewables. - Moderate economic growth. - Unequal progress across regions. | - Weak mitigation policies. - High dependence on fossil fuels. - Significant land-use changes (e.g., deforestation). - Moderate to high economic growth. | - No mitigation policies. - Very high fossil fuel use. - Accelerated land-use change (deforestation, urbanisation). - High population growth and inequality. |
| **Land Use** | Minimal deforestation: sustainable practices dominate. | Moderate deforestation: agricultural expansion stabilizes. | High deforestation due to population and energy demands. | Severe deforestation; extreme urbanisation and land conversion. |
| **Climate Extremes** | Lowest frequency and severity of extreme weather events. | Moderate frequency and severity of climate extremes. | High frequency of heatwaves, droughts, and storms. | Most severe and frequent climate extremes (e.g., heatwaves, extreme storms, sea-level rise). |

The description of SSP scenarios (e.g., SSP126, SSP245, SSP370, SSP585) follows the frameworks established by O’Neill et al. (2016), Riahi et al. (2017), and the IPCC Sixth Assessment Report (AR6, 2021).

Table S4 Model performance statistics of each algorithm type with bioclimatic and with all variables

| **Variables and Algorithms** | | | **Area under the ROC Curve (AUC)** | **True Skill Statistic (TSS)** |
| --- | --- | --- | --- | --- |
| **a. Bio-climatic only** | | |  |  |
| Regression methods | | |  |  |
|  | GLM | Generalised Linear Model | 0.86 | 0.58 |
|  | GAM | Generalised Additive Model | 0.84 | 0.55 |
|  | MARS | Multiple Adaptive Regression Splines | 0.87 | 0.61 |
|  | GBM | Generalised Boosting Model | 0.89 | 0.63 |
| Machine learning methods | | |  |  |
|  | ANN | Artificial Neural Network | 0.86 | 0.6 |
|  | RF | Random Forest | 0.97 | 0.82 |
|  | MAXENT | Maximum Entropy | 0.88 | 0.62 |
|  | SRE | Surface Range Envelope | 0.7 | 0.41 |
| Classification methods | | |  |  |
|  | CTA | Classification Tree Analysis | 0.84 | 0.6 |
|  | FDA | Flexible Discriminant Analysis | 0.86 | 0.59 |
| **b. All** | |  |  |  |
| Regression methods | | |  |  |
|  | GLM | Generalised Linear Model | 0.86 | 0.61 |
|  | GAM | Generalised Additive Model | 0.85 | 0.56 |
|  | MARS | Multiple Adaptive Regression Splines | 0.87 | 0.62 |
|  | GBM | Generalised Boosting Model | 0.89 | 0.64 |
| Machine learning methods | | |  |  |
|  | ANN | Artificial Neural Network | 0.76 | 0.45 |
|  | RF | Random Forest | 0.97 | 0.79 |
|  | MAXENT | Maximum Entropy | 0.88 | 0.62 |
|  | SRE | Surface Range Envelope | 0.68 | 0.36 |
| Classification methods | | |  |  |
|  | CTA | Classification Tree Analysis | 0.84 | 0.63 |
|  | FDA | Flexible Discriminant Analysis | 0.87 | 0.62 |


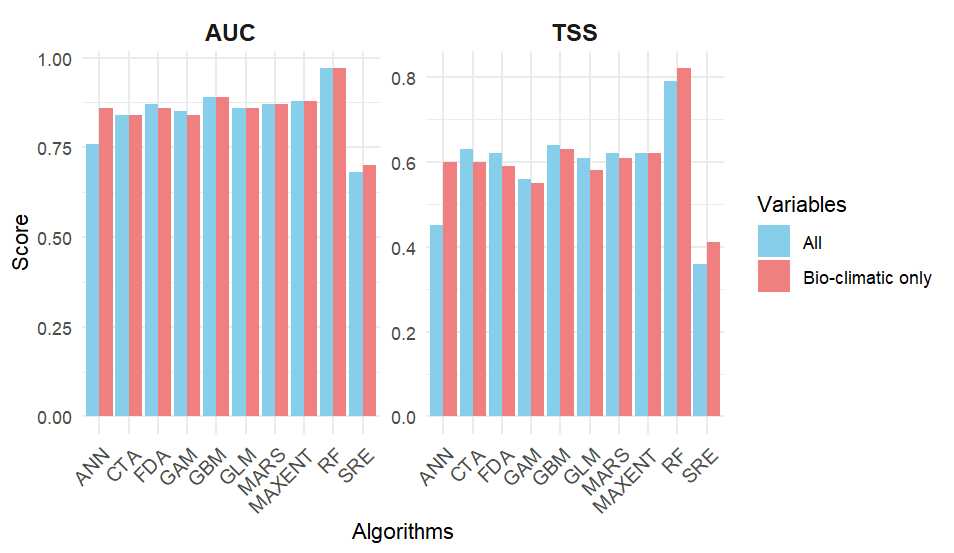


Figure S1 Model evaluation metrics (AUC and TSS) for Australian bustard (Ardeotis australis) species distribution models in BIOMOD2. Results compare bio-climatic only (red) and all-variable models (blue) across algorithms.


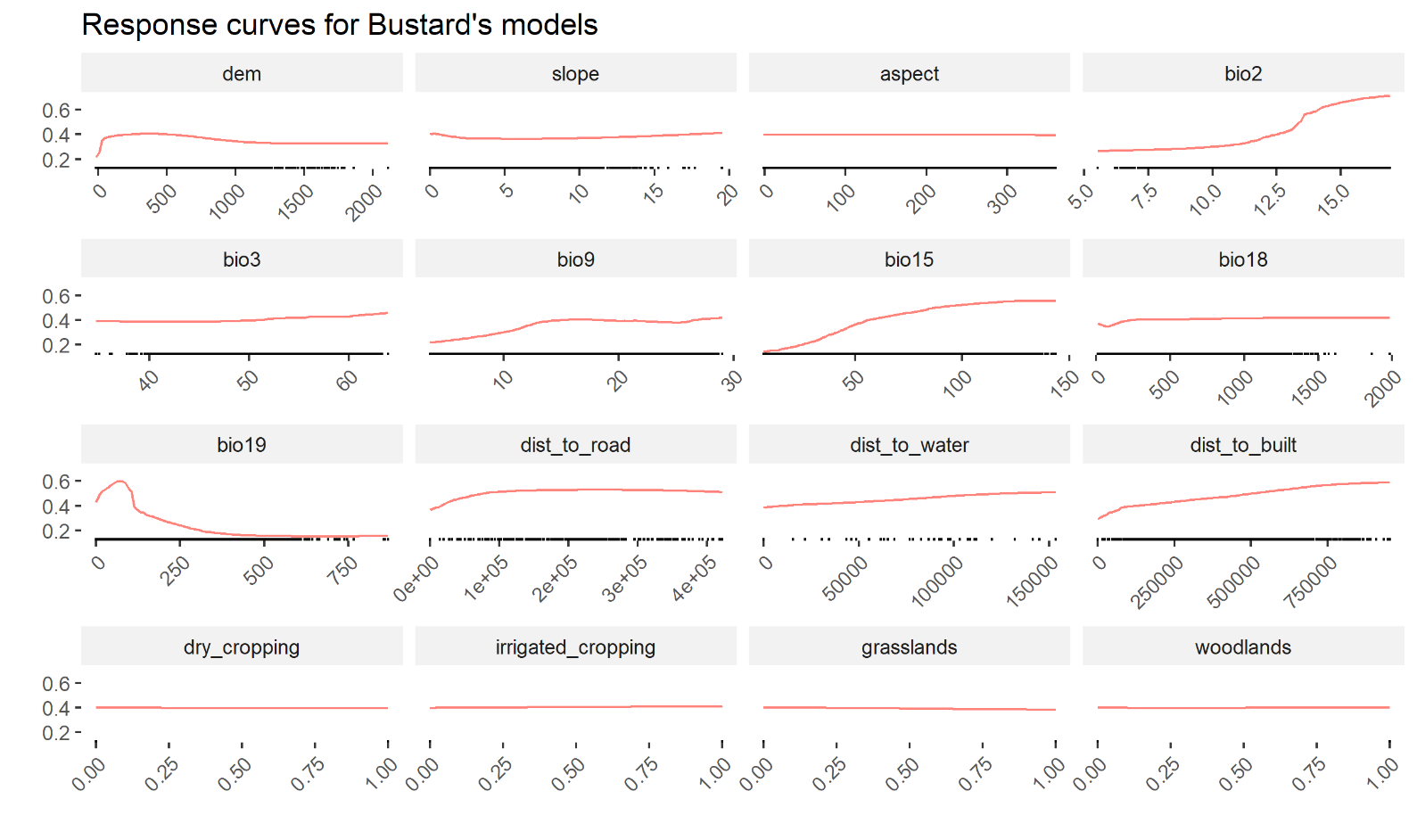


Figure S2 Response curves for Australian bustard habitat suitability across environmental variables. The y-axis represents habitat suitability, while the x-axis represents the range of each predictor variable.

Table S5 Current habitat suitability of the Australian bustard (Ardeotis australis) across Australian states, and projected changes in suitable habitat areas under different Climate Scenarios for 2050 and 2070.

| State | Suitable habitat (%) | Unsuitable habitat (%) | **SSP1-2.6 (2050)** | | | | **SSP1-2.6 (2070)** | | | | **SSP2-4.5 (2050)** | | | | **SSP2-4.5 (2070)** | | | | **SSP3-7.0 (2050)** | | | | **SSP3-7.0 (2070)** | | | | **SSP5-8.5 (2050)** | | | | **SSP5-8.5 (2070)** | | | |
| --- | --- | --- | --- | --- | --- | --- | --- | --- | --- | --- | --- | --- | --- | --- | --- | --- | --- | --- | --- | --- | --- | --- | --- | --- | --- | --- | --- | --- | --- | --- | --- | --- | --- | --- |
|  |  |  | Stable suitable | Loss | Gain | Stable unsuitable | Stable suitable | Loss | Gain | Stable unsuitable | Stable suitable | Loss | Gain | Stable unsuitable | Stable suitable | Loss | Gain | Stable unsuitable | Stable suitable | Loss | Gain | Stable unsuitable | Stable suitable | Loss | Gain | Stable unsuitable | Stable suitable | Loss | Gain | Stable unsuitable | Stable suitable | Loss | Gain | Stable unsuitable |
| New South Wales | 7 | 93 | 11 | 4 | 1 | 84 | 13 | 1 | 5 | 81 | 11 | 3 | 2 | 84 | 11 | 3 | 2 | 84 | 10 | 4 | 1 | 85 | 9 | 5 | 2 | 84 | 11 | 3 | 2 | 84 | 10 | 4 | 3 | 83 |
| Victoria | 0 | 100 | 0 | 0 | 0 | 100 | 0 | 0 | 0 | 100 | 0 | 0 | 0 | 100 | 0 | 0 | 0 | 100 | 0 | 0 | 0 | 100 | 0 | 0 | 0 | 100 | 0 | 0 | 0 | 100 | 0 | 0 | 0 | 100 |
| Queensland | 88 | 12 | 89 | 3 | 0 | 8 | 90 | 1 | 0 | 8 | 89 | 2 | 0 | 8 | 89 | 2 | 1 | 8 | 89 | 2 | 0 | 8 | 89 | 2 | 0 | 8 | 90 | 2 | 0 | 8 | 89 | 2 | 0 | 8 |
| South Australia | 45 | 55 | 45 | 20 | 0 | 35 | 53 | 10 | 0 | 37 | 49 | 13 | 0 | 37 | 45 | 13 | 9 | 34 | 49 | 14 | 0 | 37 | 46 | 17 | 0 | 37 | 48 | 15 | 0 | 37 | 48 | 15 | 1 | 37 |
| Western Australia | 86 | 14 | 87 | 3 | 0 | 10 | 89 | 1 | 1 | 9 | 88 | 2 | 0 | 10 | 89 | 1 | 1 | 9 | 88 | 2 | 0 | 10 | 88 | 2 | 1 | 9 | 88 | 2 | 0 | 10 | 89 | 1 | 1 | 9 |
| Tasmania | 0 | 100 | 0 | 0 | 0 | 100 | 0 | 0 | 0 | 100 | 0 | 0 | 0 | 100 | 0 | 0 | 0 | 100 | 0 | 0 | 0 | 100 | 0 | 0 | 0 | 100 | 0 | 0 | 0 | 100 | 0 | 0 | 0 | 100 |
| Northern Territory | 94 | 6 | 94 | 2 | 0 | 4 | 95 | 1 | 0 | 4 | 95 | 1 | 0 | 4 | 94 | 1 | 1 | 4 | 95 | 1 | 0 | 4 | 94 | 1 | 0 | 4 | 95 | 1 | 0 | 4 | 94 | 2 | 0 | 4 |
| Australian Capital | 0 | 100 | 0 | 0 | 0 | 100 | 0 | 0 | 0 | 100 | 0 | 0 | 0 | 100 | 0 | 0 | 0 | 100 | 0 | 0 | 0 | 100 | 0 | 0 | 0 | 100 | 0 | 0 | 0 | 100 | 0 | 0 | 0 | 100 |
| Other Territory | 0 | 100 | 0 | 0 | 0 | 100 | 0 | 0 | 0 | 100 | 0 | 0 | 0 | 100 | 0 | 0 | 0 | 100 | 0 | 0 | 0 | 100 | 0 | 0 | 0 | 100 | 0 | 0 | 0 | 100 | 0 | 0 | 0 | 100 |

Table S6 Current habitat suitability of the Australian bustard (Ardeotis australis) across Australian Bioregions, and projected changes in suitable habitat areas under different Climate Scenarios for 2050 and 2070.

| Bioregion | Suitable habitat (%) | Unsuitable habitat (%) | **SSP1-2.6 (2050)** | | | | **SSP1-2.6 (2070)** | | | | **SSP2-4.5 (2050)** | | | | | **SSP2-4.5 (2070)** | | | | | **SSP3-7.0 (2050)** | | | | | **SSP3-7.0 (2070)** | | | | | **SSP5-8.5 (2050)** | | | | | **SSP5-8.5 (2070)** | | | |
| --- | --- | --- | --- | --- | --- | --- | --- | --- | --- | --- | --- | --- | --- | --- | --- | --- | --- | --- | --- | --- | --- | --- | --- | --- | --- | --- | --- | --- | --- | --- | --- | --- | --- | --- | --- | --- | --- | --- | --- |
|  |  |  | Stable suitable | Loss | Gain | Stable unsuitable | Stable suitable | Loss | Gain | Stable unsuitable | Stable suitable | Loss | Gain | Stable unsuitable | Stable suitable | | Loss | Gain | Stable unsuitable | Stable suitable | | Loss | Gain | Stable unsuitable | Stable suitable | | Loss | Gain | Stable unsuitable | Stable suitable | | Loss | Gain | Stable unsuitable | Stable suitable | | Loss | Gain | Stable unsuitable |
| Arnhem_Coast | 33 | 67 | 39 | 13 | 0 | 48 | 41 | 11 | 0 | 48 | 41 | 10 | 0 | 48 | 42 | | 10 | 0 | 48 | 41 | | 10 | 0 | 48 | 39 | | 13 | 0 | 48 | 41 | | 11 | 1 | 48 | 38 | | 14 | 0 | 48 |
| Arnhem_Plateau | 80 | 20 | 84 | 12 | 0 | 4 | 86 | 10 | 0 | 4 | 86 | 10 | 0 | 4 | 89 | | 7 | 0 | 4 | 86 | | 10 | 0 | 4 | 86 | | 11 | 0 | 4 | 90 | | 6 | 0 | 3 | 85 | | 11 | 0 | 4 |
| Australian_Alps | 0 | 100 | 0 | 0 | 0 | 100 | 0 | 0 | 0 | 100 | 0 | 0 | 0 | 100 | 0 | | 0 | 0 | 100 | 0 | | 0 | 0 | 100 | 0 | | 0 | 0 | 100 | 0 | | 0 | 0 | 100 | 0 | | 0 | 0 | 100 |
| Avon_Wheatbelt | 62 | 38 | 79 | 1 | 3 | 17 | 79 | 1 | 3 | 17 | 78 | 2 | 2 | 18 | 80 | | 0 | 5 | 15 | 79 | | 1 | 4 | 16 | 80 | | 0 | 6 | 14 | 79 | | 1 | 4 | 16 | 80 | | 0 | 7 | 13 |
| Brigalow_Belt_North | 91 | 9 | 91 | 2 | 0 | 7 | 91 | 1 | 0 | 7 | 90 | 2 | 0 | 7 | 90 | | 2 | 0 | 7 | 91 | | 2 | 0 | 7 | 90 | | 2 | 0 | 7 | 91 | | 2 | 0 | 7 | 90 | | 2 | 0 | 7 |
| Brigalow_Belt_South | 72 | 28 | 77 | 1 | 2 | 20 | 77 | 0 | 5 | 17 | 76 | 1 | 3 | 20 | 76 | | 1 | 4 | 18 | 76 | | 1 | 2 | 21 | 77 | | 1 | 3 | 19 | 77 | | 0 | 3 | 19 | 76 | | 1 | 4 | 19 |
| Ben_Lomond | 0 | 100 | 0 | 0 | 0 | 100 | 0 | 0 | 0 | 100 | 0 | 0 | 0 | 100 | 0 | | 0 | 0 | 100 | 0 | | 0 | 0 | 100 | 0 | | 0 | 0 | 100 | 0 | | 0 | 0 | 100 | 0 | | 0 | 0 | 100 |
| Broken_Hill_Complex | 5 | 95 | 11 | 6 | 2 | 80 | 13 | 4 | 5 | 77 | 12 | 5 | 2 | 80 | 12 | | 6 | 3 | 79 | 12 | | 5 | 2 | 80 | 9 | | 8 | 1 | 81 | 12 | | 5 | 3 | 80 | 11 | | 6 | 3 | 79 |
| Burt_Plain | 100 | 0 | 100 | 0 | 0 | 0 | 100 | 0 | 0 | 0 | 100 | 0 | 0 | 0 | 100 | | 0 | 0 | 0 | 100 | | 0 | 0 | 0 | 100 | | 0 | 0 | 0 | 100 | | 0 | 0 | 0 | 100 | | 0 | 0 | 0 |
| Carnarvon | 94 | 6 | 96 | 1 | 0 | 3 | 96 | 1 | 0 | 3 | 96 | 1 | 0 | 3 | 96 | | 1 | 0 | 3 | 95 | | 1 | 0 | 3 | 96 | | 1 | 0 | 3 | 95 | | 1 | 0 | 3 | 96 | | 1 | 0 | 3 |
| Central_Arnhem | 91 | 9 | 93 | 3 | 0 | 4 | 94 | 2 | 0 | 4 | 94 | 2 | 0 | 4 | 94 | | 1 | 0 | 4 | 94 | | 2 | 0 | 4 | 94 | | 2 | 0 | 4 | 94 | | 1 | 0 | 4 | 94 | | 2 | 0 | 4 |
| Central_Kimberley | 100 | 0 | 100 | 0 | 0 | 0 | 100 | 0 | 0 | 0 | 100 | 0 | 0 | 0 | 100 | | 0 | 0 | 0 | 100 | | 0 | 0 | 0 | 100 | | 0 | 0 | 0 | 100 | | 0 | 0 | 0 | 100 | | 0 | 0 | 0 |
| Central_Ranges | 99 | 1 | 100 | 0 | 0 | 0 | 100 | 0 | 0 | 0 | 100 | 0 | 0 | 0 | 100 | | 0 | 0 | 0 | 100 | | 0 | 0 | 0 | 100 | | 0 | 0 | 0 | 100 | | 0 | 0 | 0 | 100 | | 0 | 0 | 0 |
| Channel_Country | 92 | 8 | 96 | 2 | 0 | 2 | 97 | 1 | 0 | 2 | 96 | 2 | 0 | 2 | 95 | | 2 | 0 | 2 | 94 | | 3 | 0 | 2 | 92 | | 6 | 0 | 2 | 95 | | 2 | 0 | 2 | 94 | | 4 | 0 | 2 |
| Central_Mackay_Coast | 14 | 86 | 14 | 13 | 0 | 73 | 16 | 11 | 0 | 73 | 14 | 13 | 0 | 73 | 13 | | 14 | 0 | 73 | 14 | | 13 | 0 | 73 | 14 | | 13 | 0 | 73 | 16 | | 11 | 0 | 73 | 13 | | 14 | 0 | 73 |
| Coolgardie | 37 | 63 | 48 | 12 | 1 | 39 | 55 | 5 | 3 | 37 | 50 | 10 | 2 | 39 | 52 | | 8 | 3 | 37 | 49 | | 11 | 2 | 38 | 50 | | 10 | 4 | 36 | 49 | | 11 | 2 | 38 | 52 | | 8 | 5 | 35 |
| Cobar_Peneplain | 0 | 100 | 0 | 0 | 1 | 99 | 1 | 0 | 3 | 96 | 0 | 0 | 2 | 98 | 0 | | 0 | 3 | 97 | 0 | | 0 | 1 | 98 | 0 | | 0 | 2 | 97 | 0 | | 0 | 3 | 97 | 0 | | 0 | 3 | 96 |
| Cape_York_Peninsula | 84 | 16 | 84 | 5 | 0 | 11 | 84 | 5 | 0 | 11 | 84 | 5 | 0 | 11 | 85 | | 4 | 0 | 11 | 84 | | 5 | 0 | 11 | 85 | | 4 | 0 | 11 | 85 | | 5 | 0 | 11 | 85 | | 4 | 0 | 11 |
| Daly_Basin | 100 | 0 | 100 | 0 | 0 | 0 | 100 | 0 | 0 | 0 | 100 | 0 | 0 | 0 | 100 | | 0 | 0 | 0 | 100 | | 0 | 0 | 0 | 100 | | 0 | 0 | 0 | 100 | | 0 | 0 | 0 | 100 | | 0 | 0 | 0 |
| Darwin_Coastal | 9 | 91 | 11 | 11 | 0 | 78 | 14 | 9 | 0 | 78 | 12 | 10 | 0 | 78 | 15 | | 7 | 0 | 78 | 12 | | 10 | 0 | 78 | 13 | | 9 | 0 | 78 | 13 | | 10 | 0 | 78 | 10 | | 12 | 0 | 78 |
| Dampierland | 96 | 4 | 96 | 2 | 0 | 2 | 96 | 2 | 0 | 2 | 96 | 2 | 0 | 2 | 96 | | 2 | 0 | 2 | 96 | | 2 | 0 | 2 | 96 | | 2 | 0 | 2 | 96 | | 2 | 0 | 2 | 96 | | 2 | 0 | 2 |
| Desert_Uplands | 100 | 0 | 100 | 0 | 0 | 0 | 100 | 0 | 0 | 0 | 100 | 0 | 0 | 0 | 100 | | 0 | 0 | 0 | 100 | | 0 | 0 | 0 | 100 | | 0 | 0 | 0 | 100 | | 0 | 0 | 0 | 100 | | 0 | 0 | 0 |
| Davenport_Murchison_Ranges | 100 | 0 | 100 | 0 | 0 | 0 | 100 | 0 | 0 | 0 | 100 | 0 | 0 | 0 | 100 | | 0 | 0 | 0 | 100 | | 0 | 0 | 0 | 100 | | 0 | 0 | 0 | 100 | | 0 | 0 | 0 | 100 | | 0 | 0 | 0 |
| Darling_Riverine_Plains | 43 | 57 | 51 | 3 | 1 | 44 | 54 | 1 | 12 | 33 | 53 | 2 | 6 | 39 | 53 | | 2 | 8 | 38 | 51 | | 4 | 4 | 42 | 51 | | 3 | 7 | 38 | 53 | | 2 | 7 | 38 | 51 | | 3 | 10 | 36 |
| Einasleigh_Uplands | 100 | 0 | 100 | 0 | 0 | 0 | 100 | 0 | 0 | 0 | 100 | 0 | 0 | 0 | 100 | | 0 | 0 | 0 | 100 | | 0 | 0 | 0 | 100 | | 0 | 0 | 0 | 100 | | 0 | 0 | 0 | 100 | | 0 | 0 | 0 |
| Esperance_Plains | 1 | 99 | 1 | 1 | 0 | 98 | 1 | 1 | 0 | 98 | 1 | 1 | 0 | 98 | 1 | | 1 | 0 | 98 | 1 | | 1 | 0 | 98 | 1 | | 1 | 0 | 98 | 1 | | 1 | 0 | 98 | 1 | | 1 | 0 | 98 |
| Eyre_Yorke_Block | 15 | 85 | 19 | 3 | 1 | 76 | 20 | 3 | 2 | 75 | 19 | 4 | 1 | 76 | 19 | | 4 | 1 | 76 | 19 | | 4 | 1 | 76 | 20 | | 2 | 2 | 75 | 17 | | 6 | 0 | 77 | 21 | | 2 | 3 | 74 |
| Finke | 100 | 0 | 100 | 0 | 0 | 0 | 100 | 0 | 0 | 0 | 100 | 0 | 0 | 0 | 100 | | 0 | 0 | 0 | 100 | | 0 | 0 | 0 | 100 | | 0 | 0 | 0 | 100 | | 0 | 0 | 0 | 100 | | 0 | 0 | 0 |
| Flinders_Lofty_Block | 6 | 94 | 7 | 6 | 0 | 87 | 8 | 4 | 1 | 87 | 8 | 5 | 1 | 87 | 8 | | 5 | 1 | 86 | 8 | | 5 | 1 | 86 | 6 | | 7 | 1 | 86 | 8 | | 5 | 0 | 87 | 7 | | 5 | 3 | 84 |
| Furneaux | 0 | 100 | 0 | 0 | 0 | 100 | 0 | 0 | 0 | 100 | 0 | 0 | 0 | 100 | 0 | | 0 | 0 | 100 | 0 | | 0 | 0 | 100 | 0 | | 0 | 0 | 100 | 0 | | 0 | 0 | 100 | 0 | | 0 | 0 | 100 |
| Gascoyne | 100 | 0 | 100 | 0 | 0 | 0 | 100 | 0 | 0 | 0 | 100 | 0 | 0 | 0 | 100 | | 0 | 0 | 0 | 100 | | 0 | 0 | 0 | 100 | | 0 | 0 | 0 | 100 | | 0 | 0 | 0 | 100 | | 0 | 0 | 0 |
| Gawler | 6 | 94 | 7 | 21 | 0 | 71 | 10 | 19 | 0 | 71 | 8 | 20 | 0 | 71 | 7 | | 21 | 0 | 71 | 9 | | 20 | 0 | 71 | 6 | | 23 | 0 | 71 | 8 | | 21 | 0 | 71 | 7 | | 22 | 0 | 71 |
| Geraldton_Sandplains | 77 | 23 | 84 | 1 | 1 | 13 | 84 | 1 | 2 | 13 | 83 | 3 | 1 | 14 | 84 | | 1 | 2 | 13 | 84 | | 2 | 1 | 14 | 84 | | 1 | 2 | 13 | 83 | | 2 | 1 | 13 | 84 | | 1 | 3 | 12 |
| Gulf_Fall_and_Uplands | 99 | 1 | 99 | 0 | 0 | 1 | 99 | 0 | 0 | 1 | 99 | 0 | 0 | 1 | 99 | | 0 | 0 | 1 | 99 | | 1 | 0 | 1 | 99 | | 0 | 0 | 1 | 99 | | 1 | 0 | 1 | 99 | | 1 | 0 | 1 |
| Gibson_Desert | 100 | 0 | 100 | 0 | 0 | 0 | 100 | 0 | 0 | 0 | 100 | 0 | 0 | 0 | 100 | | 0 | 0 | 0 | 100 | | 0 | 0 | 0 | 100 | | 0 | 0 | 0 | 100 | | 0 | 0 | 0 | 100 | | 0 | 0 | 0 |
| Great_Sandy_Desert | 100 | 0 | 100 | 0 | 0 | 0 | 100 | 0 | 0 | 0 | 100 | 0 | 0 | 0 | 100 | | 0 | 0 | 0 | 100 | | 0 | 0 | 0 | 100 | | 0 | 0 | 0 | 100 | | 0 | 0 | 0 | 100 | | 0 | 0 | 0 |
| Gulf_Coastal | 73 | 27 | 73 | 9 | 0 | 18 | 74 | 8 | 0 | 18 | 72 | 10 | 0 | 18 | 72 | | 10 | 0 | 18 | 72 | | 11 | 0 | 18 | 72 | | 11 | 0 | 18 | 71 | | 11 | 0 | 18 | 72 | | 11 | 0 | 18 |
| Gulf_Plains | 87 | 13 | 87 | 3 | 0 | 10 | 87 | 3 | 0 | 10 | 87 | 3 | 0 | 10 | 87 | | 3 | 0 | 10 | 87 | | 4 | 0 | 10 | 87 | | 3 | 0 | 10 | 87 | | 3 | 0 | 10 | 87 | | 3 | 0 | 10 |
| Great_Victoria_Desert | 80 | 20 | 82 | 9 | 0 | 9 | 84 | 6 | 0 | 9 | 81 | 9 | 0 | 9 | 82 | | 9 | 0 | 9 | 81 | | 9 | 0 | 9 | 79 | | 12 | 0 | 9 | 80 | | 10 | 0 | 9 | 81 | | 10 | 0 | 9 |
| Hampton | 48 | 52 | 50 | 27 | 0 | 23 | 52 | 25 | 0 | 23 | 50 | 27 | 0 | 23 | 49 | | 28 | 0 | 23 | 50 | | 27 | 0 | 23 | 50 | | 27 | 0 | 23 | 47 | | 30 | 0 | 23 | 51 | | 26 | 0 | 23 |
| Jarrah_Forest | 1 | 99 | 4 | 1 | 1 | 95 | 4 | 1 | 1 | 94 | 4 | 0 | 1 | 94 | 4 | | 0 | 4 | 92 | 4 | | 0 | 3 | 92 | 4 | | 0 | 6 | 89 | 4 | | 0 | 3 | 93 | 4 | | 0 | 8 | 87 |
| Kanmantoo | 0 | 100 | 0 | 0 | 0 | 100 | 0 | 0 | 0 | 100 | 0 | 0 | 0 | 100 | 0 | | 0 | 0 | 100 | 0 | | 0 | 0 | 100 | 0 | | 0 | 0 | 100 | 0 | | 0 | 0 | 100 | 0 | | 0 | 0 | 100 |
| King | 0 | 100 | 0 | 0 | 0 | 100 | 0 | 0 | 0 | 100 | 0 | 0 | 0 | 100 | 0 | | 0 | 0 | 100 | 0 | | 0 | 0 | 100 | 0 | | 0 | 0 | 100 | 0 | | 0 | 0 | 100 | 0 | | 0 | 0 | 100 |
| Little_Sandy_Desert | 100 | 0 | 100 | 0 | 0 | 0 | 100 | 0 | 0 | 0 | 100 | 0 | 0 | 0 | 100 | | 0 | 0 | 0 | 100 | | 0 | 0 | 0 | 100 | | 0 | 0 | 0 | 100 | | 0 | 0 | 0 | 100 | | 0 | 0 | 0 |
| MacDonnell_Ranges | 100 | 0 | 100 | 0 | 0 | 0 | 100 | 0 | 0 | 0 | 100 | 0 | 0 | 0 | 100 | | 0 | 0 | 0 | 100 | | 0 | 0 | 0 | 100 | | 0 | 0 | 0 | 100 | | 0 | 0 | 0 | 100 | | 0 | 0 | 0 |
| Mallee | 17 | 83 | 25 | 9 | 2 | 65 | 28 | 6 | 4 | 62 | 26 | 7 | 2 | 64 | 29 | | 5 | 6 | 60 | 25 | | 9 | 2 | 64 | 27 | | 6 | 6 | 61 | 27 | | 7 | 2 | 64 | 29 | | 5 | 8 | 58 |
| Murray_Darling_Depression | 2 | 98 | 4 | 2 | 0 | 94 | 3 | 2 | 0 | 94 | 3 | 3 | 0 | 94 | 3 | | 3 | 1 | 94 | 2 | | 4 | 0 | 94 | 3 | | 3 | 0 | 94 | 2 | | 4 | 0 | 94 | 3 | | 3 | 1 | 93 |
| Mitchell_Grass_Downs | 100 | 0 | 100 | 0 | 0 | 0 | 100 | 0 | 0 | 0 | 100 | 0 | 0 | 0 | 100 | | 0 | 0 | 0 | 100 | | 0 | 0 | 0 | 100 | | 0 | 0 | 0 | 100 | | 0 | 0 | 0 | 100 | | 0 | 0 | 0 |
| Mount_Isa_Inlier | 100 | 0 | 100 | 0 | 0 | 0 | 100 | 0 | 0 | 0 | 100 | 0 | 0 | 0 | 100 | | 0 | 0 | 0 | 100 | | 0 | 0 | 0 | 100 | | 0 | 0 | 0 | 100 | | 0 | 0 | 0 | 100 | | 0 | 0 | 0 |
| Mulga_Lands | 66 | 34 | 72 | 9 | 0 | 19 | 78 | 3 | 4 | 15 | 73 | 8 | 0 | 19 | 74 | | 7 | 0 | 19 | 69 | | 12 | 0 | 19 | 68 | | 13 | 0 | 19 | 72 | | 9 | 0 | 19 | 69 | | 12 | 0 | 19 |
| Murchison | 92 | 8 | 95 | 4 | 0 | 1 | 98 | 1 | 0 | 1 | 96 | 3 | 0 | 1 | 96 | | 3 | 0 | 1 | 96 | | 2 | 0 | 1 | 95 | | 3 | 0 | 1 | 94 | | 4 | 0 | 1 | 95 | | 4 | 0 | 1 |
| Nandewar | 0 | 100 | 1 | 0 | 2 | 97 | 1 | 0 | 6 | 93 | 1 | 0 | 4 | 96 | 1 | | 0 | 6 | 93 | 1 | | 0 | 2 | 98 | 1 | | 0 | 4 | 96 | 1 | | 0 | 5 | 94 | 1 | | 0 | 4 | 95 |
| Naracoorte_Coastal_Plain | 0 | 100 | 0 | 0 | 0 | 100 | 0 | 0 | 0 | 100 | 0 | 0 | 0 | 100 | 0 | | 0 | 0 | 100 | 0 | | 0 | 0 | 100 | 0 | | 0 | 0 | 100 | 0 | | 0 | 0 | 100 | 0 | | 0 | 0 | 100 |
| New_England_Tablelands | 0 | 100 | 0 | 0 | 0 | 100 | 0 | 0 | 0 | 100 | 0 | 0 | 0 | 100 | 0 | | 0 | 0 | 100 | 0 | | 0 | 0 | 100 | 0 | | 0 | 0 | 100 | 0 | | 0 | 0 | 100 | 0 | | 0 | 0 | 100 |
| NSW_North_Coast | 0 | 100 | 0 | 0 | 0 | 100 | 0 | 0 | 0 | 100 | 0 | 0 | 0 | 100 | 0 | | 0 | 0 | 100 | 0 | | 0 | 0 | 100 | 0 | | 0 | 0 | 100 | 0 | | 0 | 0 | 100 | 0 | | 0 | 0 | 100 |
| Northern_Kimberley | 93 | 7 | 93 | 1 | 0 | 6 | 93 | 1 | 0 | 6 | 93 | 1 | 0 | 6 | 93 | | 1 | 0 | 6 | 93 | | 1 | 0 | 6 | 93 | | 1 | 0 | 6 | 93 | | 1 | 0 | 6 | 93 | | 1 | 0 | 6 |
| NSW_South_Western_Slopes | 0 | 100 | 0 | 0 | 0 | 100 | 0 | 0 | 0 | 100 | 0 | 0 | 0 | 100 | 0 | | 0 | 0 | 100 | 0 | | 0 | 0 | 100 | 0 | | 0 | 0 | 100 | 0 | | 0 | 0 | 100 | 0 | | 0 | 0 | 100 |
| Nullarbor | 97 | 3 | 98 | 2 | 0 | 0 | 98 | 1 | 0 | 0 | 98 | 2 | 0 | 0 | 98 | | 2 | 0 | 0 | 98 | | 2 | 0 | 0 | 98 | | 2 | 0 | 0 | 98 | | 2 | 0 | 0 | 98 | | 1 | 0 | 0 |
| Ord_Victoria_Plain | 100 | 0 | 100 | 0 | 0 | 0 | 100 | 0 | 0 | 0 | 100 | 0 | 0 | 0 | 100 | | 0 | 0 | 0 | 100 | | 0 | 0 | 0 | 100 | | 0 | 0 | 0 | 100 | | 0 | 0 | 0 | 100 | | 0 | 0 | 0 |
| Pine_Creek | 81 | 19 | 85 | 5 | 0 | 10 | 86 | 3 | 0 | 10 | 85 | 5 | 0 | 10 | 85 | | 5 | 0 | 10 | 85 | | 5 | 0 | 10 | 82 | | 7 | 0 | 10 | 84 | | 5 | 0 | 10 | 82 | | 8 | 0 | 10 |
| Pilbara | 99 | 1 | 99 | 0 | 0 | 1 | 99 | 0 | 0 | 1 | 99 | 0 | 0 | 1 | 99 | | 0 | 0 | 1 | 99 | | 0 | 0 | 1 | 99 | | 0 | 0 | 1 | 99 | | 0 | 0 | 1 | 99 | | 0 | 0 | 1 |
| Riverina | 0 | 100 | 0 | 0 | 0 | 100 | 0 | 0 | 0 | 99 | 0 | 0 | 0 | 100 | 0 | | 0 | 0 | 99 | 0 | | 0 | 0 | 99 | 0 | | 0 | 0 | 100 | 0 | | 0 | 0 | 99 | 0 | | 0 | 0 | 99 |
| South_East_Coastal_Plain | 0 | 100 | 0 | 0 | 0 | 100 | 0 | 0 | 0 | 100 | 0 | 0 | 0 | 100 | 0 | | 0 | 0 | 100 | 0 | | 0 | 0 | 100 | 0 | | 0 | 0 | 100 | 0 | | 0 | 0 | 100 | 0 | | 0 | 0 | 100 |
| South_East_Corner | 0 | 100 | 0 | 0 | 0 | 100 | 0 | 0 | 0 | 100 | 0 | 0 | 0 | 100 | 0 | | 0 | 0 | 100 | 0 | | 0 | 0 | 100 | 0 | | 0 | 0 | 100 | 0 | | 0 | 0 | 100 | 0 | | 0 | 0 | 100 |
| South_Eastern_Highlands | 0 | 100 | 0 | 0 | 0 | 100 | 0 | 0 | 0 | 100 | 0 | 0 | 0 | 100 | 0 | | 0 | 0 | 100 | 0 | | 0 | 0 | 100 | 0 | | 0 | 0 | 100 | 0 | | 0 | 0 | 100 | 0 | | 0 | 0 | 100 |
| South_Eastern_Queensland | 8 | 92 | 12 | 3 | 0 | 85 | 14 | 1 | 1 | 84 | 11 | 5 | 0 | 85 | 11 | | 5 | 0 | 85 | 11 | | 4 | 0 | 85 | 13 | | 3 | 0 | 85 | 13 | | 2 | 0 | 84 | 11 | | 4 | 0 | 85 |
| Simpson_Strzelecki_Dunefields | 75 | 25 | 80 | 11 | 0 | 8 | 84 | 8 | 0 | 8 | 82 | 9 | 0 | 8 | 80 | | 12 | 0 | 8 | 80 | | 12 | 0 | 8 | 78 | | 14 | 0 | 8 | 81 | | 11 | 0 | 8 | 80 | | 12 | 0 | 8 |
| Stony_Plains | 58 | 42 | 61 | 27 | 0 | 12 | 75 | 13 | 0 | 12 | 67 | 22 | 0 | 12 | 65 | | 23 | 0 | 12 | 69 | | 19 | 0 | 12 | 56 | | 32 | 0 | 12 | 62 | | 26 | 0 | 12 | 65 | | 24 | 0 | 12 |
| Sturt_Plateau | 100 | 0 | 100 | 0 | 0 | 0 | 100 | 0 | 0 | 0 | 100 | 0 | 0 | 0 | 100 | | 0 | 0 | 0 | 100 | | 0 | 0 | 0 | 100 | | 0 | 0 | 0 | 100 | | 0 | 0 | 0 | 100 | | 0 | 0 | 0 |
| Southern_Volcanic_Plain | 0 | 100 | 0 | 0 | 0 | 100 | 0 | 0 | 0 | 100 | 0 | 0 | 0 | 100 | 0 | | 0 | 0 | 100 | 0 | | 0 | 0 | 100 | 0 | | 0 | 0 | 100 | 0 | | 0 | 0 | 100 | 0 | | 0 | 0 | 100 |
| Swan_Coastal_Plain | 6 | 94 | 15 | 1 | 2 | 83 | 15 | 1 | 2 | 82 | 14 | 1 | 1 | 83 | 15 | | 0 | 2 | 83 | 15 | | 1 | 2 | 83 | 15 | | 0 | 3 | 82 | 15 | | 0 | 2 | 83 | 15 | | 0 | 3 | 81 |
| Sydney_Basin | 0 | 100 | 0 | 0 | 0 | 100 | 0 | 0 | 0 | 100 | 0 | 0 | 0 | 100 | 0 | | 0 | 0 | 100 | 0 | | 0 | 0 | 100 | 0 | | 0 | 0 | 100 | 0 | | 0 | 0 | 100 | 0 | | 0 | 1 | 99 |
| Tanami | 100 | 0 | 100 | 0 | 0 | 0 | 100 | 0 | 0 | 0 | 100 | 0 | 0 | 0 | 100 | | 0 | 0 | 0 | 100 | | 0 | 0 | 0 | 100 | | 0 | 0 | 0 | 100 | | 0 | 0 | 0 | 100 | | 0 | 0 | 0 |
| Tasmanian_Central_Highlands | 0 | 100 | 0 | 0 | 0 | 100 | 0 | 0 | 0 | 100 | 0 | 0 | 0 | 100 | 0 | | 0 | 0 | 100 | 0 | | 0 | 0 | 100 | 0 | | 0 | 0 | 100 | 0 | | 0 | 0 | 100 | 0 | | 0 | 0 | 100 |
| Tiwi_Cobourg | 31 | 69 | 39 | 16 | 0 | 45 | 41 | 14 | 0 | 45 | 42 | 13 | 0 | 45 | 46 | | 9 | 0 | 45 | 43 | | 12 | 0 | 45 | 38 | | 17 | 0 | 45 | 45 | | 10 | 0 | 45 | 36 | | 19 | 0 | 45 |
| Tasmanian_Northern_Midlands | 0 | 100 | 0 | 0 | 0 | 100 | 0 | 0 | 0 | 100 | 0 | 0 | 0 | 100 | 0 | | 0 | 0 | 100 | 0 | | 0 | 0 | 100 | 0 | | 0 | 0 | 100 | 0 | | 0 | 0 | 100 | 0 | | 0 | 0 | 100 |
| Tasmanian_Northern_Slopes | 0 | 100 | 0 | 0 | 0 | 100 | 0 | 0 | 0 | 100 | 0 | 0 | 0 | 100 | 0 | | 0 | 0 | 100 | 0 | | 0 | 0 | 100 | 0 | | 0 | 0 | 100 | 0 | | 0 | 0 | 100 | 0 | | 0 | 0 | 100 |
| Tasmanian_South_East | 0 | 100 | 0 | 0 | 0 | 100 | 0 | 0 | 0 | 100 | 0 | 0 | 0 | 100 | 0 | | 0 | 0 | 100 | 0 | | 0 | 0 | 100 | 0 | | 0 | 0 | 100 | 0 | | 0 | 0 | 100 | 0 | | 0 | 0 | 100 |
| Tasmanian_Southern_Ranges | 0 | 100 | 0 | 0 | 0 | 100 | 0 | 0 | 0 | 100 | 0 | 0 | 0 | 100 | 0 | | 0 | 0 | 100 | 0 | | 0 | 0 | 100 | 0 | | 0 | 0 | 100 | 0 | | 0 | 0 | 100 | 0 | | 0 | 0 | 100 |
| Tasmanian_West | 0 | 100 | 0 | 0 | 0 | 100 | 0 | 0 | 0 | 100 | 0 | 0 | 0 | 100 | 0 | | 0 | 0 | 100 | 0 | | 0 | 0 | 100 | 0 | | 0 | 0 | 100 | 0 | | 0 | 0 | 100 | 0 | | 0 | 0 | 100 |
| Victoria_Bonaparte | 91 | 9 | 90 | 3 | 0 | 7 | 91 | 2 | 0 | 7 | 90 | 3 | 0 | 7 | 91 | | 2 | 0 | 7 | 90 | | 3 | 0 | 7 | 90 | | 3 | 0 | 7 | 90 | | 3 | 0 | 7 | 89 | | 3 | 0 | 7 |
| Victorian_Midlands | 0 | 100 | 0 | 0 | 0 | 100 | 0 | 0 | 0 | 100 | 0 | 0 | 0 | 100 | 0 | | 0 | 0 | 100 | 0 | | 0 | 0 | 100 | 0 | | 0 | 0 | 100 | 0 | | 0 | 0 | 100 | 0 | | 0 | 0 | 100 |
| Warren | 0 | 100 | 0 | 0 | 0 | 100 | 0 | 0 | 0 | 100 | 0 | 0 | 0 | 100 | 0 | | 0 | 0 | 100 | 0 | | 0 | 0 | 100 | 0 | | 0 | 0 | 100 | 0 | | 0 | 0 | 100 | 0 | | 0 | 0 | 100 |
| Wet_Tropics | 19 | 81 | 19 | 4 | 0 | 77 | 19 | 4 | 0 | 77 | 19 | 4 | 0 | 77 | 18 | | 5 | 0 | 77 | 19 | | 5 | 0 | 77 | 18 | | 5 | 0 | 77 | 19 | | 5 | 0 | 77 | 18 | | 5 | 0 | 77 |
| Yalgoo | 94 | 6 | 96 | 1 | 0 | 3 | 96 | 1 | 0 | 3 | 96 | 1 | 0 | 3 | 96 | | 1 | 0 | 3 | 96 | | 1 | 0 | 3 | 96 | | 1 | 0 | 3 | 96 | | 1 | 0 | 3 | 96 | | 1 | 0 | 3 |

Table S7 Current Habitat Suitability of the Australian bustard (Ardeotis australis) across Australian Indigenous Protected Areas

| Indigenous Protected Area | Suitable habitat (%) | Unsuitable habitat (%) | **SSP1-2.6 (2050)** | | | | **SSP1-2.6 (2070)** | | | | **SSP2-4.5 (2050)** | | | | **SSP2-4.5 (2070)** | | | | **SSP3-7.0 (2050)** | | | | **SSP3-7.0 (2070)** | | | | **SSP5-8.5 (2050)** | | | | **SSP5-8.5 (2070)** | | | |
| --- | --- | --- | --- | --- | --- | --- | --- | --- | --- | --- | --- | --- | --- | --- | --- | --- | --- | --- | --- | --- | --- | --- | --- | --- | --- | --- | --- | --- | --- | --- | --- | --- | --- | --- |
|  |  |  | Stable suitable | Loss | Gain | Stable unsuitable | Stable suitable | Loss | Gain | Stable unsuitable | Stable suitable | Loss | Gain | Stable unsuitable | Stable suitable | Loss | Gain | Stable unsuitable | Stable suitable | Loss | Gain | Stable unsuitable | Stable suitable | Loss | Gain | Stable unsuitable | Stable suitable | Loss | Gain | Stable unsuitable | Stable suitable | Loss | Gain | Stable unsuitable |
| Ngurra Kayanta | 100 | 0 | 100 | 0 | 0 | 0 | 100 | 0 | 0 | 0 | 100 | 0 | 0 | 0 | 100 | 0 | 0 | 0 | 100 | 0 | 0 | 0 | 100 | 0 | 0 | 0 | 100 | 0 | 0 | 0 | 100 | 0 | 0 | 0 |
| Wuthathi | 76 | 24 | 78 | 7 | 0 | 16 | 74 | 6 | 4 | 15 | 77 | 7 | 0 | 16 | 77 | 7 | 0 | 16 | 77 | 7 | 0 | 16 | 77 | 7 | 0 | 16 | 78 | 7 | 0 | 16 | 78 | 6 | 0 | 16 |
| Anangu Tjutaku | 100 | 0 | 100 | 0 | 0 | 0 | 100 | 0 | 0 | 0 | 100 | 0 | 0 | 0 | 100 | 0 | 0 | 0 | 100 | 0 | 0 | 0 | 100 | 0 | 0 | 0 | 100 | 0 | 0 | 0 | 100 | 0 | 0 | 0 |
| Angas Downs | 100 | 0 | 100 | 0 | 0 | 0 | 100 | 0 | 0 | 0 | 100 | 0 | 0 | 0 | 100 | 0 | 0 | 0 | 100 | 0 | 0 | 0 | 100 | 0 | 0 | 0 | 100 | 0 | 0 | 0 | 100 | 0 | 0 | 0 |
| Angkum | 76 | 24 | 78 | 3 | 0 | 19 | 78 | 2 | 1 | 19 | 76 | 5 | 0 | 19 | 79 | 2 | 0 | 19 | 76 | 5 | 0 | 19 | 79 | 2 | 0 | 19 | 78 | 3 | 0 | 19 | 79 | 2 | 0 | 19 |
| Anindilyakwa | 33 | 67 | 37 | 15 | 0 | 48 | 35 | 12 | 9 | 44 | 38 | 14 | 0 | 48 | 40 | 12 | 0 | 48 | 38 | 14 | 0 | 48 | 43 | 9 | 0 | 48 | 38 | 13 | 0 | 48 | 47 | 4 | 0 | 48 |
| Bardi Jawi | 69 | 31 | 64 | 15 | 0 | 20 | 59 | 13 | 9 | 19 | 66 | 14 | 0 | 20 | 66 | 14 | 0 | 20 | 65 | 15 | 0 | 20 | 65 | 14 | 0 | 20 | 64 | 15 | 0 | 20 | 65 | 15 | 0 | 20 |
| Dambimangari | 82 | 18 | 83 | 1 | 0 | 17 | 82 | 1 | 0 | 17 | 83 | 1 | 0 | 17 | 83 | 1 | 0 | 17 | 83 | 1 | 0 | 17 | 83 | 1 | 0 | 17 | 83 | 1 | 0 | 16 | 83 | 1 | 0 | 17 |
| Deen Maar | 0 | 100 | 0 | 0 | 0 | 100 | 0 | 0 | 0 | 100 | 0 | 0 | 0 | 100 | 0 | 0 | 0 | 100 | 0 | 0 | 0 | 100 | 0 | 0 | 0 | 100 | 0 | 0 | 0 | 100 | 0 | 0 | 0 | 100 |
| Dhimurru | 63 | 37 | 67 | 4 | 0 | 30 | 64 | 4 | 3 | 29 | 65 | 5 | 0 | 30 | 66 | 5 | 0 | 30 | 65 | 5 | 0 | 30 | 64 | 6 | 0 | 30 | 65 | 5 | 0 | 30 | 66 | 5 | 0 | 30 |
| Djelk | 12 | 88 | 21 | 22 | 1 | 56 | 20 | 18 | 13 | 49 | 24 | 19 | 2 | 55 | 31 | 12 | 2 | 55 | 26 | 17 | 2 | 55 | 27 | 16 | 1 | 56 | 34 | 9 | 3 | 54 | 24 | 19 | 0 | 57 |
| Djelk - Stage 2 | 0 | 100 | 0 | 0 | 0 | 100 | 0 | 0 | 0 | 100 | 0 | 0 | 0 | 100 | 0 | 0 | 0 | 100 | 0 | 0 | 0 | 100 | 0 | 0 | 0 | 100 | 0 | 0 | 0 | 100 | 0 | 0 | 0 | 100 |
| Framlingham Fore | 40 | 60 | 41 | 3 | 0 | 56 | 41 | 3 | 2 | 55 | 42 | 3 | 0 | 56 | 41 | 3 | 0 | 56 | 41 | 3 | 0 | 56 | 41 | 3 | 0 | 56 | 41 | 3 | 0 | 56 | 40 | 4 | 0 | 56 |
| Ganalanga-Mindib | 0 | 100 | 0 | 0 | 0 | 100 | 0 | 0 | 0 | 100 | 0 | 0 | 0 | 100 | 0 | 0 | 0 | 100 | 0 | 0 | 0 | 100 | 0 | 0 | 0 | 100 | 0 | 0 | 0 | 100 | 0 | 0 | 0 | 100 |
| Girringun | 100 | 0 | 100 | 0 | 0 | 0 | 100 | 0 | 0 | 0 | 100 | 0 | 0 | 0 | 100 | 0 | 0 | 0 | 100 | 0 | 0 | 0 | 100 | 0 | 0 | 0 | 100 | 0 | 0 | 0 | 100 | 0 | 0 | 0 |
| Great Dog Island | 0 | 100 | 0 | 0 | 0 | 100 | 0 | 0 | 0 | 100 | 0 | 0 | 0 | 100 | 0 | 0 | 0 | 100 | 0 | 0 | 0 | 100 | 0 | 0 | 0 | 100 | 0 | 0 | 0 | 100 | 0 | 0 | 0 | 100 |
| Guanaba | 0 | 100 | 0 | 0 | 0 | 100 | 0 | 0 | 0 | 100 | 0 | 0 | 0 | 100 | 0 | 0 | 0 | 100 | 0 | 0 | 0 | 100 | 0 | 0 | 0 | 100 | 0 | 0 | 0 | 100 | 0 | 0 | 0 | 100 |
| Jamba Dhandan Du | 0 | 100 | 0 | 0 | 0 | 100 | 0 | 0 | 0 | 100 | 0 | 0 | 0 | 100 | 0 | 0 | 0 | 100 | 0 | 0 | 0 | 100 | 0 | 0 | 0 | 100 | 0 | 0 | 0 | 100 | 0 | 0 | 0 | 100 |
| Kalka – Pipalyat | 97 | 3 | 100 | 0 | 0 | 0 | 100 | 0 | 0 | 0 | 100 | 0 | 0 | 0 | 100 | 0 | 0 | 0 | 99 | 1 | 0 | 0 | 100 | 0 | 0 | 0 | 100 | 0 | 0 | 0 | 100 | 0 | 0 | 0 |
| Antara - Sandy B | 100 | 0 | 100 | 0 | 0 | 0 | 100 | 0 | 0 | 0 | 100 | 0 | 0 | 0 | 100 | 0 | 0 | 0 | 100 | 0 | 0 | 0 | 100 | 0 | 0 | 0 | 100 | 0 | 0 | 0 | 100 | 0 | 0 | 0 |
| Apara – Makiri | 100 | 0 | 100 | 0 | 0 | 0 | 100 | 0 | 0 | 0 | 100 | 0 | 0 | 0 | 100 | 0 | 0 | 0 | 100 | 0 | 0 | 0 | 100 | 0 | 0 | 0 | 100 | 0 | 0 | 0 | 100 | 0 | 0 | 0 |
| Babel Island | 0 | 100 | 0 | 0 | 0 | 100 | 0 | 0 | 0 | 100 | 0 | 0 | 0 | 100 | 0 | 2 | 0 | 100 | 0 | 0 | 0 | 100 | 0 | 8 | 0 | 100 | 100 | 0 | 0 | 100 | 0 | 0 | 0 | 100 |
| Badger Island | 0 | 100 | 0 | 0 | 0 | 100 | 0 | 0 | 0 | 100 | 0 | 0 | 0 | 100 | 0 | 0 | 0 | 100 | 0 | 0 | 0 | 100 | 0 | 0 | 0 | 100 | 0 | 0 | 0 | 100 | 0 | 0 | 0 | 100 |
| Balanggarra | 0 | 100 | 0 | 0 | 0 | 100 | 0 | 0 | 0 | 100 | 0 | 0 | 0 | 100 | 0 | 0 | 0 | 100 | 0 | 0 | 0 | 100 | 0 | 0 | 0 | 100 | 0 | 0 | 0 | 100 | 0 | 0 | 0 | 100 |
| Birriliburu | 0 | 100 | 0 | 0 | 0 | 100 | 0 | 0 | 0 | 100 | 0 | 0 | 0 | 100 | 0 | 0 | 0 | 100 | 0 | 0 | 0 | 100 | 0 | 0 | 0 | 100 | 0 | 0 | 0 | 100 | 0 | 0 | 0 | 100 |
| Boorabee And The | 97 | 3 | 97 | 1 | 0 | 2 | 97 | 1 | 1 | 2 | 97 | 1 | 0 | 2 | 98 | 1 | 0 | 2 | 97 | 1 | 0 | 2 | 97 | 1 | 0 | 2 | 97 | 1 | 0 | 2 | 97 | 1 | 0 | 2 |
| Brewarrina Ngemb | 100 | 0 | 100 | 0 | 0 | 0 | 100 | 0 | 0 | 0 | 100 | 0 | 0 | 0 | 100 | 0 | 0 | 0 | 100 | 0 | 0 | 0 | 100 | 0 | 0 | 0 | 100 | 0 | 0 | 0 | 100 | 0 | 0 | 0 |
| Crocodile Island | 0 | 100 | 0 | 0 | 0 | 100 | 0 | 0 | 0 | 100 | 0 | 0 | 0 | 100 | 0 | 0 | 0 | 100 | 0 | 0 | 0 | 100 | 0 | 0 | 0 | 100 | 0 | 0 | 0 | 100 | 0 | 0 | 0 | 100 |
| Risdon Cove | 0 | 100 | 0 | 0 | 0 | 100 | 0 | 0 | 0 | 100 | 0 | 0 | 0 | 100 | 0 | 0 | 0 | 100 | 0 | 0 | 0 | 100 | 0 | 0 | 0 | 100 | 0 | 0 | 0 | 100 | 0 | 0 | 0 | 100 |
| South-East Arnhe | 1 | 99 | 2 | 5 | 0 | 92 | 2 | 5 | 3 | 89 | 3 | 5 | 0 | 92 | 5 | 3 | 0 | 92 | 4 | 3 | 0 | 92 | 2 | 5 | 0 | 92 | 5 | 3 | 0 | 92 | 2 | 6 | 0 | 92 |
| Tarriwa Kurrukun | 0 | 100 | 0 | 0 | 0 | 100 | 0 | 0 | 0 | 100 | 0 | 0 | 0 | 100 | 0 | 0 | 0 | 100 | 0 | 0 | 0 | 100 | 0 | 0 | 0 | 100 | 0 | 0 | 0 | 100 | 0 | 0 | 0 | 100 |
| Thuwathu Bujimul | 79 | 21 | 80 | 5 | 0 | 15 | 79 | 4 | 3 | 15 | 80 | 5 | 0 | 15 | 80 | 5 | 0 | 15 | 79 | 6 | 0 | 15 | 81 | 4 | 0 | 15 | 79 | 5 | 0 | 15 | 81 | 4 | 0 | 15 |
| Uunguu | 100 | 0 | 100 | 0 | 0 | 0 | 100 | 0 | 0 | 0 | 100 | 0 | 0 | 0 | 100 | 0 | 0 | 0 | 100 | 0 | 0 | 0 | 100 | 0 | 0 | 0 | 100 | 0 | 0 | 0 | 100 | 0 | 0 | 0 |
| Toogimbie | 0 | 100 | 0 | 0 | 0 | 100 | 0 | 0 | 0 | 100 | 0 | 0 | 0 | 100 | 0 | 0 | 0 | 100 | 0 | 0 | 0 | 100 | 0 | 0 | 0 | 100 | 0 | 0 | 0 | 100 | 0 | 0 | 0 | 100 |
| Tyrendarra | 4 | 96 | 3 | 11 | 0 | 86 | 4 | 10 | 7 | 80 | 4 | 10 | 0 | 86 | 4 | 10 | 0 | 86 | 4 | 10 | 0 | 86 | 4 | 10 | 0 | 86 | 4 | 10 | 0 | 86 | 5 | 10 | 0 | 86 |
| Walalkara | 83 | 17 | 83 | 2 | 0 | 15 | 82 | 2 | 1 | 15 | 83 | 2 | 0 | 15 | 83 | 2 | 0 | 15 | 83 | 2 | 0 | 15 | 83 | 1 | 0 | 15 | 83 | 2 | 0 | 15 | 83 | 2 | 0 | 15 |
| Wardang Island | 0 | 100 | 0 | 0 | 0 | 100 | 0 | 0 | 0 | 100 | 0 | 0 | 0 | 100 | 0 | 0 | 0 | 100 | 0 | 0 | 0 | 100 | 0 | 0 | 0 | 100 | 0 | 0 | 0 | 100 | 0 | 0 | 0 | 100 |
| Wardaman | 0 | 100 | 0 | 0 | 0 | 100 | 0 | 0 | 0 | 100 | 0 | 0 | 0 | 100 | 0 | 0 | 0 | 100 | 0 | 0 | 0 | 100 | 0 | 0 | 0 | 100 | 0 | 0 | 0 | 100 | 0 | 0 | 0 | 100 |
| Warddeken | 97 | 3 | 97 | 3 | 0 | 0 | 100 | 0 | 0 | 0 | 97 | 3 | 0 | 0 | 97 | 3 | 0 | 0 | 98 | 2 | 0 | 0 | 93 | 7 | 0 | 0 | 97 | 3 | 0 | 0 | 97 | 3 | 0 | 0 |
| Karajarri | 0 | 100 | 0 | 0 | 0 | 100 | 0 | 0 | 0 | 100 | 0 | 0 | 0 | 100 | 0 | 0 | 0 | 100 | 0 | 0 | 0 | 100 | 0 | 0 | 0 | 100 | 0 | 0 | 0 | 100 | 0 | 0 | 0 | 100 |
| Katiti Petermann | 100 | 0 | 100 | 0 | 0 | 0 | 100 | 0 | 0 | 0 | 100 | 0 | 0 | 0 | 100 | 0 | 0 | 0 | 100 | 0 | 0 | 0 | 100 | 0 | 0 | 0 | 100 | 0 | 0 | 0 | 100 | 0 | 0 | 0 |
| Kiwirrkurra | 84 | 16 | 86 | 10 | 0 | 3 | 83 | 8 | 6 | 3 | 88 | 8 | 0 | 3 | 92 | 5 | 0 | 3 | 89 | 8 | 0 | 3 | 87 | 10 | 0 | 3 | 92 | 5 | 1 | 3 | 88 | 9 | 0 | 3 |
| Kurtonitj | 0 | 100 | 0 | 0 | 0 | 100 | 0 | 0 | 0 | 100 | 0 | 0 | 0 | 100 | 0 | 0 | 0 | 100 | 0 | 0 | 0 | 100 | 0 | 0 | 0 | 100 | 0 | 0 | 0 | 100 | 0 | 0 | 0 | 100 |
| Lake Condah | 0 | 100 | 0 | 0 | 0 | 0 | 0 | 0 | 0 | 100 | 0 | 0 | 0 | 100 | 0 | 0 | 0 | 100 | 0 | 0 | 0 | 100 | 0 | 0 | 0 | 100 | 0 | 0 | 0 | 100 | 0 | 0 | 0 | 100 |
| Laynhapuy | 100 | 0 | 100 | 0 | 0 | 0 | 100 | 0 | 0 | 0 | 100 | 0 | 0 | 0 | 100 | 0 | 0 | 0 | 100 | 0 | 0 | 0 | 100 | 0 | 0 | 0 | 100 | 0 | 0 | 0 | 100 | 0 | 0 | 0 |
| Lungatalanana | 0 | 100 | 0 | 0 | 0 | 100 | 0 | 0 | 0 | 100 | 0 | 0 | 0 | 100 | 0 | 0 | 0 | 100 | 0 | 0 | 0 | 100 | 0 | 0 | 0 | 100 | 0 | 0 | 0 | 100 | 0 | 0 | 0 | 100 |
| Mandingalbay Yid | 0 | 100 | 0 | 0 | 0 | 100 | 0 | 0 | 0 | 100 | 0 | 0 | 0 | 100 | 0 | 0 | 0 | 100 | 0 | 0 | 0 | 100 | 0 | 0 | 0 | 100 | 0 | 0 | 0 | 100 | 0 | 0 | 0 | 100 |
| Marri-Jabin Tha | 27 | 73 | 33 | 15 | 0 | 52 | 31 | 13 | 9 | 48 | 35 | 13 | 0 | 52 | 40 | 8 | 0 | 52 | 36 | 12 | 0 | 52 | 44 | 4 | 1 | 52 | 39 | 8 | 0 | 52 | 37 | 11 | 0 | 52 |
| Marthakal | 0 | 100 | 0 | 0 | 0 | 100 | 0 | 0 | 0 | 100 | 0 | 0 | 0 | 100 | 0 | 0 | 0 | 100 | 0 | 0 | 0 | 100 | 0 | 0 | 0 | 100 | 0 | 0 | 0 | 100 | 0 | 0 | 0 | 100 |
| Martu | 9 | 91 | 9 | 14 | 0 | 77 | 8 | 13 | 9 | 71 | 9 | 14 | 0 | 78 | 9 | 14 | 0 | 78 | 8 | 15 | 0 | 78 | 8 | 15 | 0 | 78 | 8 | 15 | 0 | 78 | 7 | 16 | 0 | 78 |
| Matuwa And Kurra | 1 | 99 | 2 | 22 | 0 | 76 | 7 | 14 | 10 | 69 | 1 | 22 | 0 | 76 | 11 | 12 | 0 | 76 | 1 | 23 | 0 | 76 | 10 | 13 | 1 | 76 | 3 | 21 | 0 | 76 | 7 | 16 | 4 | 73 |
| Mawonga | 35 | 65 | 37 | 7 | 0 | 54 | 36 | 7 | 5 | 53 | 40 | 5 | 0 | 55 | 38 | 7 | 0 | 55 | 40 | 5 | 0 | 55 | 36 | 9 | 0 | 55 | 39 | 6 | 0 | 55 | 36 | 9 | 0 | 55 |
| Minyumai | 100 | 0 | 100 | 0 | 0 | 0 | 100 | 0 | 0 | 0 | 100 | 0 | 0 | 0 | 100 | 0 | 0 | 0 | 100 | 0 | 0 | 0 | 100 | 0 | 0 | 0 | 100 | 0 | 0 | 0 | 100 | 0 | 0 | 0 |
| Mount Chappell I | 0 | 100 | 0 | 0 | 0 | 100 | 0 | 0 | 0 | 100 | 0 | 0 | 0 | 100 | 0 | 0 | 0 | 100 | 0 | 0 | 0 | 100 | 0 | 0 | 0 | 100 | 0 | 0 | 0 | 100 | 0 | 0 | 0 | 100 |
| Mount Willoughby | 0 | 100 | 0 | 0 | 0 | 100 | 0 | 0 | 0 | 100 | 0 | 0 | 0 | 100 | 0 | 0 | 0 | 100 | 0 | 0 | 0 | 100 | 0 | 0 | 0 | 100 | 0 | 0 | 0 | 100 | 0 | 0 | 0 | 100 |
| Nantawarrina | 0 | 100 | 0 | 0 | 0 | 100 | 0 | 0 | 0 | 100 | 0 | 0 | 0 | 100 | 0 | 0 | 0 | 100 | 0 | 0 | 0 | 100 | 0 | 0 | 0 | 100 | 0 | 0 | 0 | 100 | 0 | 0 | 0 | 100 |
| Ngaanyatjarra | 0 | 100 | 0 | 0 | 0 | 100 | 0 | 0 | 0 | 100 | 0 | 0 | 0 | 100 | 0 | 0 | 0 | 100 | 0 | 0 | 0 | 100 | 0 | 0 | 0 | 100 | 0 | 0 | 0 | 100 | 0 | 0 | 0 | 100 |
| Ngadju | 36 | 64 | 38 | 60 | 0 | 2 | 52 | 27 | 19 | 2 | 38 | 60 | 0 | 2 | 40 | 58 | 0 | 2 | 47 | 51 | 0 | 2 | 25 | 73 | 0 | 2 | 42 | 56 | 0 | 2 | 48 | 50 | 0 | 2 |
| Ngunya Jargoon | 12 | 88 | 19 | 13 | 0 | 68 | 30 | 3 | 2 | 65 | 28 | 4 | 3 | 65 | 21 | 11 | 1 | 67 | 29 | 3 | 4 | 64 | 9 | 23 | 0 | 68 | 23 | 9 | 1 | 67 | 20 | 12 | 0 | 68 |
| Ngururrpa | 100 | 0 | 100 | 0 | 0 | 0 | 100 | 0 | 0 | 0 | 100 | 0 | 0 | 0 | 100 | 0 | 0 | 0 | 100 | 0 | 0 | 0 | 100 | 0 | 0 | 0 | 100 | 0 | 0 | 0 | 100 | 0 | 0 | 0 |
| Nijinda Durlga | 32 | 68 | 36 | 18 | 0 | 46 | 45 | 7 | 5 | 43 | 42 | 12 | 1 | 46 | 44 | 9 | 2 | 44 | 35 | 19 | 1 | 46 | 37 | 17 | 3 | 44 | 38 | 16 | 1 | 45 | 43 | 11 | 4 | 42 |
| Ninghan | 0 | 100 | 0 | 0 | 0 | 100 | 0 | 0 | 0 | 100 | 0 | 0 | 0 | 100 | 0 | 0 | 0 | 100 | 0 | 0 | 0 | 100 | 0 | 0 | 0 | 100 | 0 | 0 | 0 | 100 | 0 | 0 | 0 | 100 |
| Northern Tanami | 100 | 0 | 100 | 0 | 0 | 0 | 100 | 0 | 0 | 0 | 100 | 0 | 0 | 0 | 100 | 0 | 0 | 0 | 100 | 0 | 0 | 0 | 100 | 0 | 0 | 0 | 100 | 0 | 0 | 0 | 100 | 0 | 0 | 0 |
| Nyangumarta Warr | 0 | 100 | 0 | 2 | 0 | 98 | 0 | 2 | 1 | 97 | 0 | 2 | 0 | 98 | 0 | 2 | 0 | 98 | 0 | 2 | 0 | 98 | 0 | 2 | 0 | 98 | 0 | 2 | 0 | 98 | 0 | 2 | 0 | 98 |
| Olkola | 73 | 27 | 91 | 0 | 4 | 5 | 95 | 0 | 0 | 4 | 90 | 1 | 2 | 7 | 91 | 0 | 5 | 4 | 91 | 0 | 3 | 5 | 91 | 0 | 6 | 3 | 91 | 0 | 4 | 5 | 91 | 0 | 8 | 1 |
| Paruku | 100 | 0 | 100 | 0 | 0 | 0 | 100 | 0 | 0 | 0 | 100 | 0 | 0 | 0 | 100 | 0 | 0 | 0 | 100 | 0 | 0 | 0 | 100 | 0 | 0 | 0 | 100 | 0 | 0 | 0 | 100 | 0 | 0 | 0 |
| Preminghana | 100 | 0 | 100 | 0 | 0 | 0 | 100 | 0 | 0 | 0 | 100 | 0 | 0 | 0 | 100 | 0 | 0 | 0 | 100 | 0 | 0 | 0 | 100 | 0 | 0 | 0 | 100 | 0 | 0 | 0 | 100 | 0 | 0 | 0 |
| Yappala | 100 | 0 | 100 | 0 | 0 | 0 | 100 | 0 | 0 | 0 | 100 | 0 | 0 | 0 | 100 | 0 | 0 | 0 | 100 | 0 | 0 | 0 | 100 | 0 | 0 | 0 | 100 | 0 | 0 | 0 | 100 | 0 | 0 | 0 |
| Yawuru | 100 | 0 | 100 | 0 | 0 | 0 | 100 | 0 | 0 | 0 | 100 | 0 | 0 | 0 | 100 | 0 | 0 | 0 | 100 | 0 | 0 | 0 | 100 | 0 | 0 | 0 | 100 | 0 | 0 | 0 | 100 | 0 | 0 | 0 |
| Talaroo | 0 | 100 | 0 | 0 | 0 | 100 | 0 | 0 | 0 | 100 | 0 | 0 | 0 | 100 | 0 | 0 | 0 | 100 | 0 | 0 | 0 | 100 | 0 | 0 | 0 | 100 | 0 | 0 | 0 | 100 | 0 | 0 | 0 | 100 |
| Mayala | 0 | 100 | 0 | 0 | 0 | 100 | 0 | 0 | 0 | 100 | 0 | 0 | 0 | 100 | 0 | 0 | 0 | 100 | 0 | 0 | 0 | 100 | 0 | 0 | 0 | 100 | 0 | 0 | 0 | 100 | 0 | 0 | 0 | 100 |
| Nyul Nyul | 82 | 18 | 83 | 10 | 0 | 6 | 84 | 6 | 4 | 6 | 84 | 9 | 0 | 6 | 81 | 12 | 0 | 6 | 82 | 12 | 0 | 6 | 84 | 9 | 0 | 6 | 83 | 11 | 0 | 6 | 80 | 13 | 0 | 6 |
| Warlu Jilajaa Ju | 100 | 0 | 100 | 0 | 0 | 0 | 100 | 0 | 0 | 0 | 100 | 0 | 0 | 0 | 100 | 0 | 0 | 0 | 100 | 0 | 0 | 0 | 100 | 0 | 0 | 0 | 100 | 0 | 0 | 0 | 100 | 0 | 0 | 0 |
| Warraberalgal An | 3 | 97 | 3 | 0 | 0 | 97 | 3 | 0 | 0 | 97 | 3 | 0 | 0 | 97 | 3 | 0 | 0 | 97 | 3 | 0 | 0 | 97 | 3 | 0 | 0 | 97 | 3 | 0 | 0 | 97 | 3 | 0 | 0 | 97 |
| Watarru | 85 | 15 | 82 | 11 | 0 | 7 | 76 | 10 | 7 | 6 | 82 | 11 | 0 | 7 | 82 | 11 | 0 | 7 | 82 | 11 | 0 | 7 | 82 | 11 | 0 | 7 | 82 | 11 | 0 | 7 | 81 | 12 | 0 | 7 |
| Wattleridge | 100 | 0 | 100 | 0 | 0 | 0 | 100 | 0 | 0 | 0 | 100 | 0 | 0 | 0 | 100 | 0 | 0 | 0 | 100 | 0 | 0 | 0 | 100 | 0 | 0 | 0 | 100 | 0 | 0 | 0 | 100 | 0 | 0 | 0 |
| Weilmoringle | 0 | 100 | 0 | 0 | 0 | 100 | 0 | 0 | 0 | 100 | 0 | 0 | 0 | 100 | 0 | 0 | 0 | 100 | 0 | 0 | 0 | 100 | 0 | 0 | 0 | 100 | 0 | 0 | 0 | 100 | 0 | 0 | 0 | 100 |
| Wik | 100 | 0 | 100 | 0 | 0 | 0 | 100 | 0 | 0 | 0 | 100 | 0 | 0 | 0 | 100 | 0 | 0 | 0 | 100 | 0 | 0 | 0 | 100 | 0 | 0 | 0 | 100 | 0 | 0 | 0 | 100 | 0 | 0 | 0 |
| Wilinggin | 0 | 100 | 0 | 0 | 0 | 100 | 0 | 0 | 0 | 100 | 0 | 0 | 0 | 100 | 0 | 0 | 0 | 100 | 0 | 0 | 0 | 100 | 0 | 0 | 0 | 100 | 0 | 0 | 0 | 100 | 0 | 0 | 0 | 100 |
| Yalata | 93 | 7 | 100 | 0 | 0 | 0 | 100 | 0 | 0 | 0 | 100 | 0 | 0 | 0 | 100 | 0 | 0 | 0 | 95 | 5 | 0 | 0 | 100 | 0 | 0 | 0 | 100 | 0 | 0 | 0 | 100 | 0 | 0 | 0 |
| Yanyuwa Barni | 27 | 73 | 22 | 23 | 0 | 55 | 20 | 19 | 13 | 48 | 22 | 23 | 0 | 55 | 24 | 20 | 0 | 55 | 26 | 19 | 0 | 55 | 25 | 20 | 0 | 55 | 21 | 24 | 0 | 55 | 32 | 13 | 0 | 55 |

|  | **2050** | **2070** |
| --- | --- | --- |
| SSP1-2.6 |  | 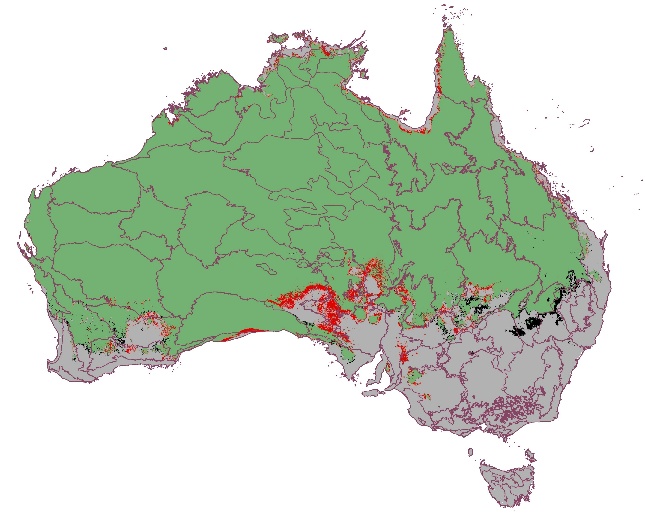 |
| SSP2-4.5 | 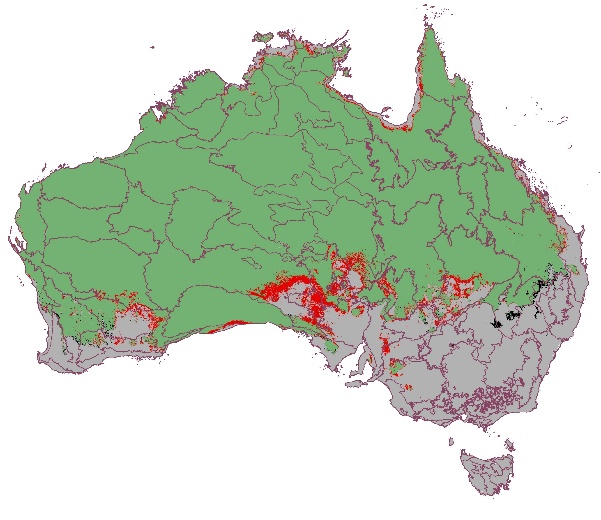 | 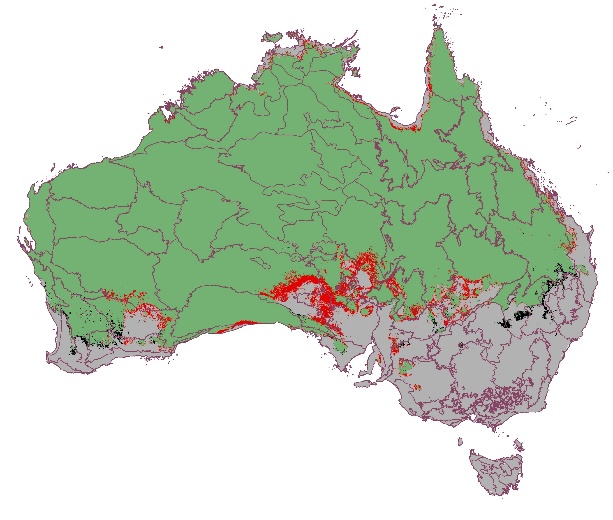 |
| SSP3-7.0 | 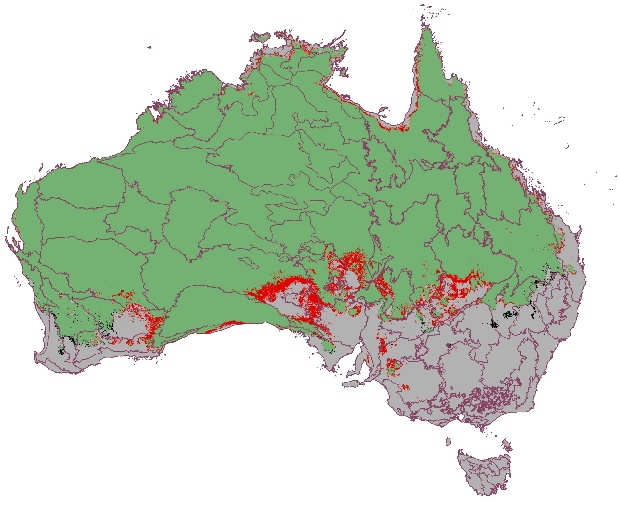 | 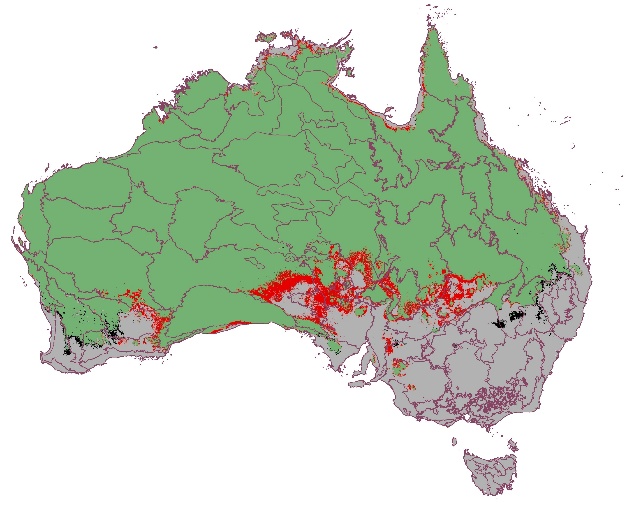 |
| SSP5-8.5 | 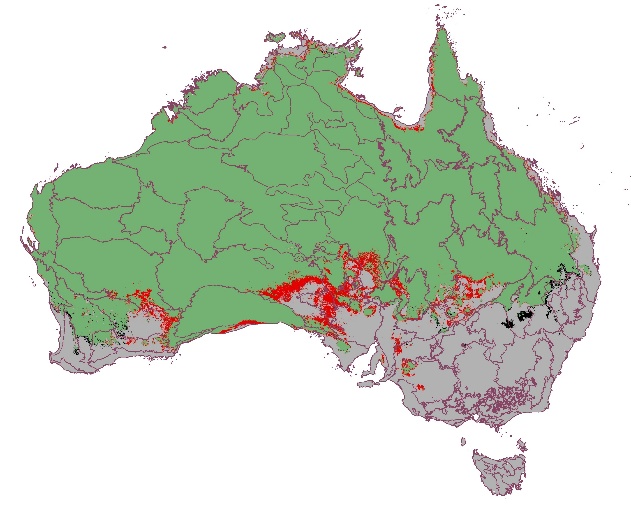 | 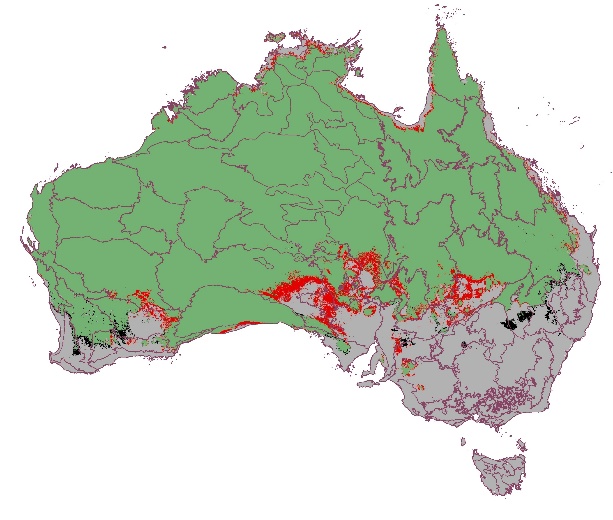 |
| Legend | \|  \|  \|  \|  \| \| --- \| --- \| --- \| --- \| \| Stable suitable habitat \| Loss habitat \| Gain habitat \| Stable unsuitable habitat \| | |

Figure S3 Projected habitat suitability for the Australian bustard (Ardeotis australis) within Bioregions for 2050 and 2070 under different climatic scenarios

|  | **2050** | **2070** |
| --- | --- | --- |
| SSP1-2.6 |  | 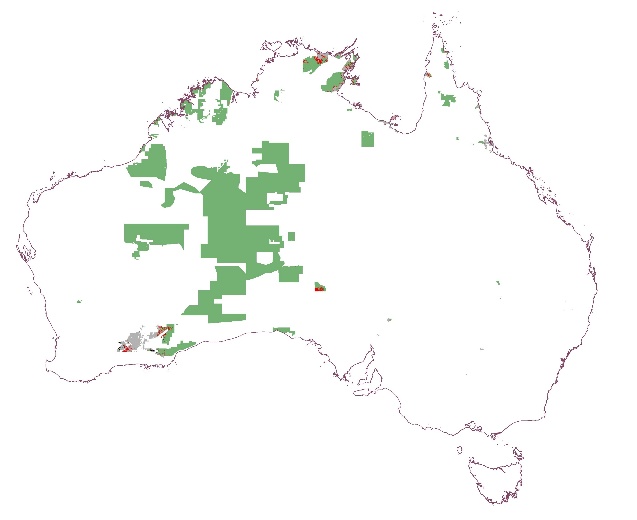 |
| SSP2-4.5 | 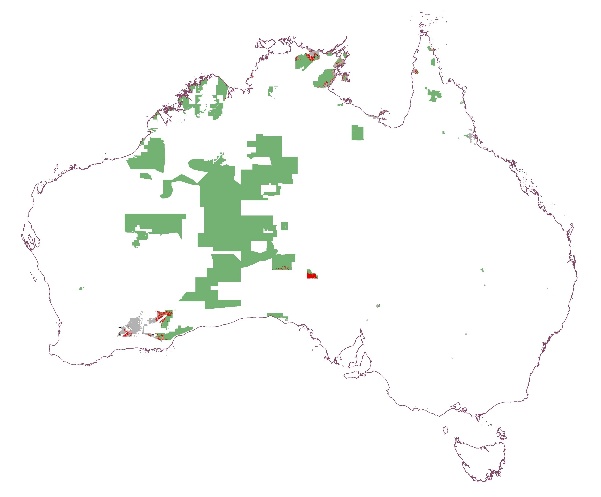 | 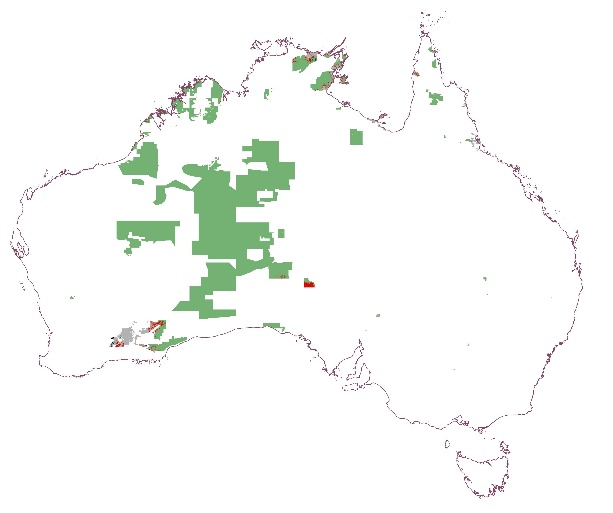 |
| SSP3-7.0 | 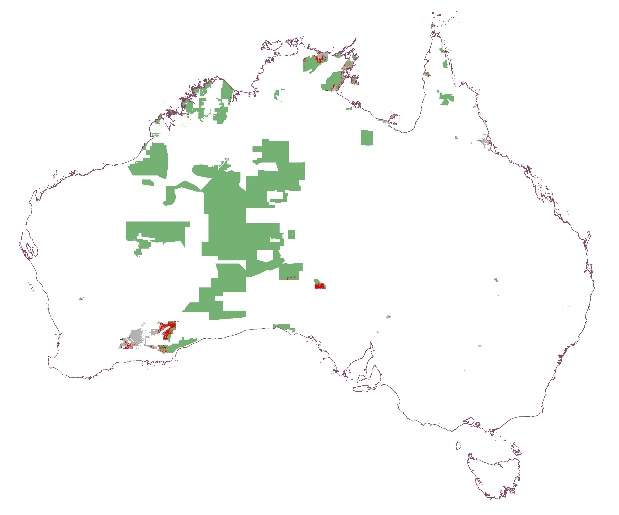 | 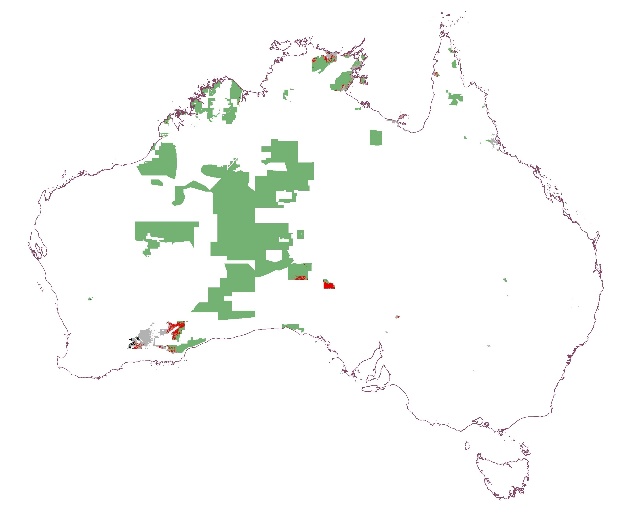 |
| SSP5-8.5 | 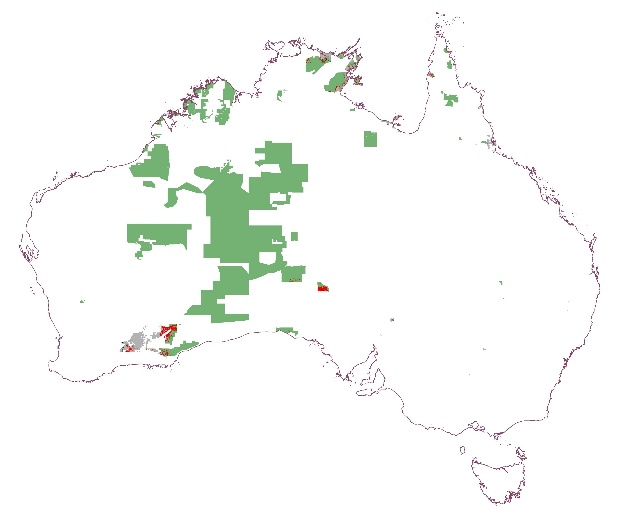 | 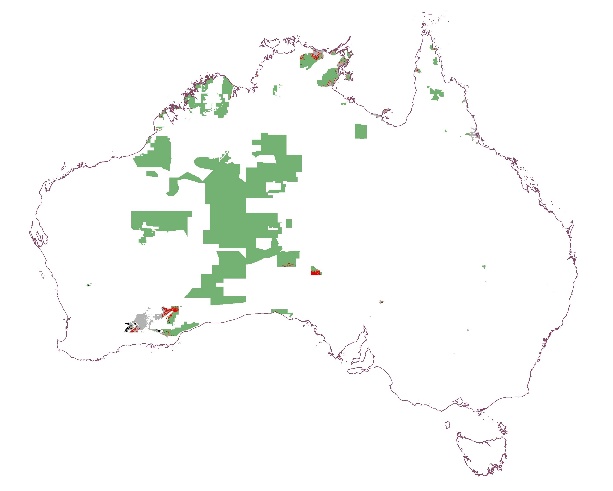 |
| Legend | \|  \|  \|  \|  \| \| --- \| --- \| --- \| --- \| \| Stable suitable habitat \| Loss habitat \| Gain habitat \| Stable unsuitable habitat \| | |

Figure S4 Projected habitat suitability for the Australian bustard (Ardeotis australis) within IPAs for 2050 and 2070 under different climatic scenarios
